# Tet Methylcytosine Dioxygenase 3 Promotes Cardiovascular Senescence by DNA 5‐Hydroxymethylcytosine‐Mediated Sp1 Transcription Factor Expression

**DOI:** 10.1002/mco2.70261

**Published:** 2025-06-19

**Authors:** Yanqi Dang, Jing Ma, Shuang Ling, Shurong Wang, Huining Guo, Jun Liu, Guang Ji, Jin‐Wen Xu

**Affiliations:** ^1^ Institute of Interdisciplinary Medical Science Shanghai University of Traditional Chinese Medicine Shanghai China; ^2^ Institute of Digestive Diseases China‐Canada Center of Research for Digestive Diseases (ccCRDD) Longhua Hospital Shanghai University of Traditional Chinese Medicine Shanghai China; ^3^ State Key Laboratory of Integration and Innovation of Classic Formula and Modern Chinese Medicine Shanghai University of Traditional Chinese Medicine Shanghai China; ^4^ Seventh People's Hospital of Shanghai University of Traditional Chinese Medicine Shanghai China; ^5^ School of Integrative Medicine Shanghai University of Traditional Chinese Medicine Shanghai China

**Keywords:** cellular senescence, DNA 5‐hydroxymethylcytosine, Sp1 transcription factor, Tet methylcytosine dioxygenase 3, tumor protein p53

## Abstract

Cellular senescence is a significant contributor to various age‐related diseases. Tet methylcytosine dioxygenase 3 (TET3) is a pivotal regulator of epigenetic modifications, and this study aimed to elucidate its role in cellular senescence. The study utilized replication and paraquat (PQ)‐induced senescent endothelial cells, as well as TET3 heterozygous, p53 heterozygous, and PQ‐induced senescent mice as experimental models. Senescent endothelial cells were analyzed using hydromethylated DNA immunoprecipitation sequencing, β‐galactosidase staining, real‐time PCR, western blotting, immunofluorescence staining, dot blot, chromatin immunoprecipitation assay, and luciferase reporter assays. These analyses were conducted following TET3 knockdown and gene overexpression. TET3 is instrumental in the elevation of 5‐hydroxymethylcytosine (5‐hmC) levels in both replication and PQ‐induced senescent endothelial cells, as well as in the cardiovascular systems of PQ‐induced aging mice. TET3 significantly promoted cellular senescence in PQ‐induced endothelial cells and mice. TET3 facilitates the upregulation of the Sp1 transcription factor (SP1) through 5‐hmC modification, leading to a synergistic interaction between SP1 and ETS proto‐oncogene 1 that further enhances p53 expression. Moreover, p53 not only promotes cellular senescence in vitro and in vivo but also reciprocally enhances TET3 and 5‐hmC levels. These findings underscore the critical role of elevated TET3 and 5‐hmC levels in cellular senescence.

## Graphical abstract

TET3 was identified to be a key DNA‐active demethylase responsible for regulating cardiovascular senescence. TET3 facilitates the upregulation of SP1 through 5‐hmC modification, leading to a synergistic interaction between SP1 and ETS‐1 that further enhances p53 expression. Moreover, p53 not only promotes cellular senescence in vitro and in vivo but also reciprocally enhances TET3 and 5‐hmC levels.

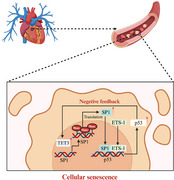

## 1 Introduction

Aging is characterized by twelve distinct features, among which epigenetic alterations are particularly significant [1]. Epigenetic modifications are integral to mammalian aging, and the restoration of epigenomic integrity has been proposed as a potential strategy to reverse the aging process [2]. DNA methylation status has been utilized as a vital indicator of cell senescence, and the epigenetic clock, which relies on DNA methylation patterns, is extensively employed in aging and cancer research. Within the framework of epigenetic regulation, which includes the “writing,” “erasing,” and “reading” functions of epigenetic regulation, the specific role of DNA demethylase ten‐eleven translocation (TET) enzyme family in aging remains to be fully elucidated. The TET family comprises three subtypes of methylcytosine dioxygenase, including TET1, TET2, and TET3, each of which is involved in various physiological and pathological processes. The importance of TET1 in the development of embryonic stem cells has been proven [3, 4, 5], whereas TET2 has been shown to be pivotal in the progression of inflammatory diseases [6, 7], atherosclerosis [8, 9], diabetes [10], clonal hematopoiesis [8, 11], and cancer [10, 12]. TET3 has been implicated in promoting the growth of acute myeloid leukemia and the activation of leukemia stem cell‐associated pathways [13], as well as in the upregulation of 5‐hydroxymethylcytosine (5‐hmC) levels in synovitis tissues of rheumatoid arthritis [14]. Studies have reported increased levels of TET2 and TET3 in the cerebellum, but not in the frontal cortex of aging mice [15]. In contrast, in aging mouse oocytes, all DNA demethylation marks, including 5‐hmC, 5‐formylcytosine, and 5‐carboxycytosine, are dynamically regulated, a process associated with elevated TET3 expression [16].

Cellular senescence is a prevalent phenomenon within blood vessels, particularly in endothelial cells, and is associated with a range of cardiovascular disorders, including atherosclerosis, hypertension, and diabetes [17, 18, 19]. In patients with acute coronary syndrome, endothelial microparticles can initiate premature senescence in coronary artery endothelial cells, leading to an elevation in their thrombogenicity [20]. Senescent endothelial cells are characterized by the increased activity and secretion of proinflammatory cytokines, which are pivotal in the pathophysiology of arterial dysfunction and various cardiovascular disorders [21]. Upon exposure to deleterious stimuli, endothelial cells undergo genetic and epigenetic dysfunction, leading to cell cycle arrest and cellular senescence [22, 23]. Research has shown that vascular endothelial cellular senescence contributes to heart failure with preserved ejection fraction [24, 25, 26], disruption of the blood–brain barrier [27], and development of pulmonary hypertension [28].

Replicative senescence is identified as a major contributor to endothelial cell senescence [21]. Although there is a recognized correlation between 5hmC and aging as well as age‐related diseases, the specific role of TET3 in cardiovascular aging remains largely unknown. Furthermore, studies have demonstrated that paraquat (PQ) may also induce cellular senescence [29, 30]. PQ increases oxidative stress in the heart and promotes the senescent phenotype of cardiomyocytes [31, 32].

Consequently, this research utilized replicative senescent and PQ‐induced senescent endothelial cells, as well as the cardiovascular system in PQ‐induced aging mice, to explore the role of TET3 and 5‐hmC. The results demonstrated a significant elevation in the levels of 5‐hmC and TET3 in replicative senescent and PQ‐induced senescent endothelial cells, as well as in the cardiovascular tissues of aging mice. TET3 was identified as a critical DNA demethylase involved in the regulation of cardiovascular senescence. Further investigation revealed that TET3 regulated the level of Sp1 transcription factor (SP1) through 5‐hmC modification, and SP1 further regulated p53 expression through cooperative interactions with ETS proto‐oncogene 1 (ETS‐1). These findings highlight the specific role of TET3 in cellular senescence and cardiovascular aging.

## 2 Results

### 2.1 Increased 5‐hmC Levels in Senescent Endothelial Cells and PQ‐Induced Aging Mice

**FIGURE 1:**
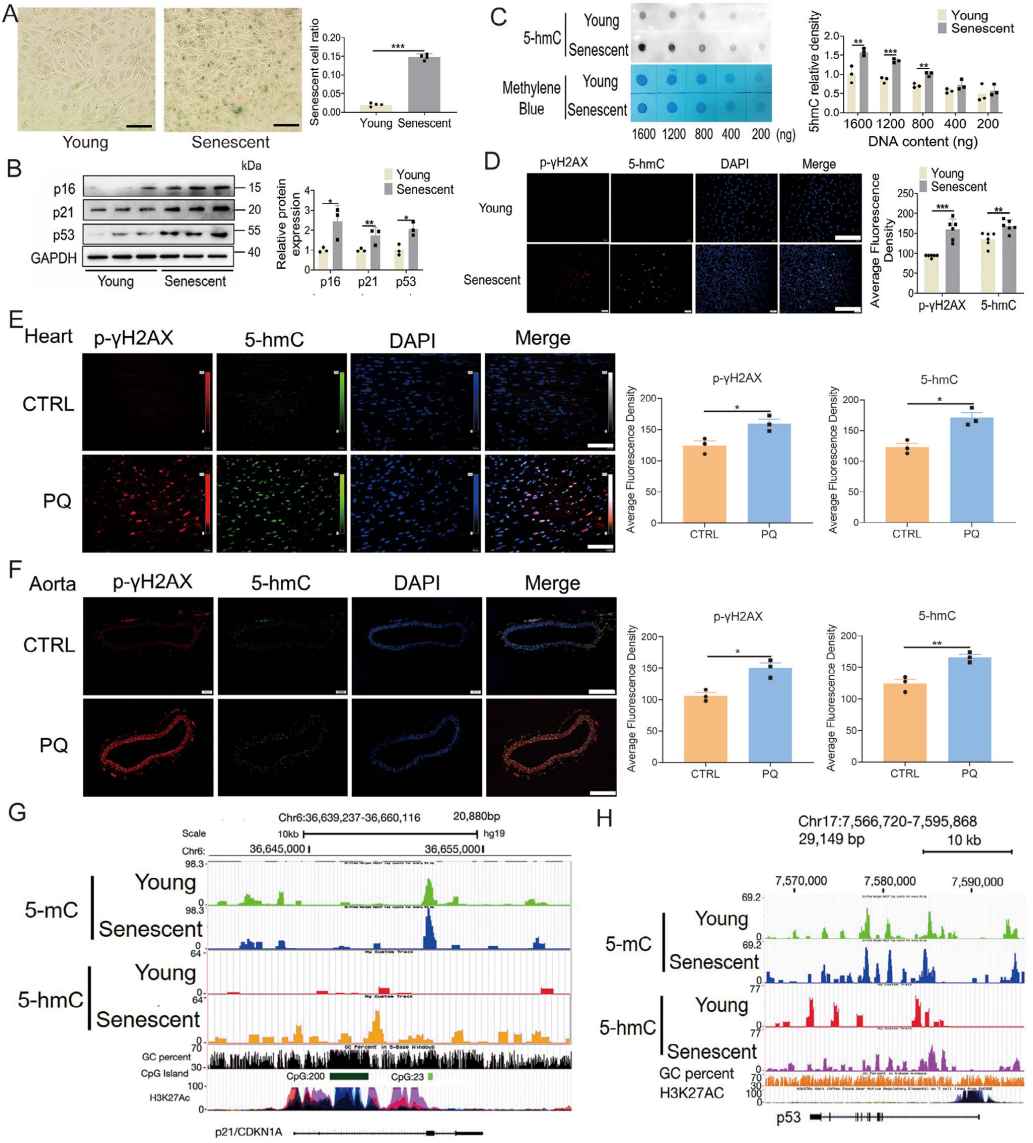
5‐hmC levels in senescent HUVECs and PQ‐induced aging mice. (A) β‐galactosidase staining and senescent cell ratio in replicative senescent HUVECs (*n* = 4 for each group, unpaired two‐sided *t*‐test, scale bars = 100 µm). (B) Protein levels of p16, p21, and p53 in replicative senescent HUVECs (*n* = 3 for each group, unpaired two‐sided *t*‐test). (C) 5‐hmC levels in replicative senescent HUVECs assayed by using a dot blot; methylene blue staining used as an internal control of the DNA content of samples (*n* = 3 for each group, unpaired two‐sided *t*‐test). (D) Levels of p‐γH2AX and 5‐hmC in replicative senescent HUVECs (*n* = 6 for each group, unpaired two‐sided *t*‐test, scale bars = 100 µm). Levels of p‐γH2AX and 5‐hmC in the heart (E, scale bars = 100 µm) and aorta tissue (F, scale bars = 400 µm) of PQ‐treated mice (*n* = 3 for each group, unpaired two‐sided *t*‐test). (G) 5‐mC and 5‐hmC landscape changes of the p21 in the senescent cells compared with the young cells. (H) 5‐mC and 5‐hmC landscape changes in the p53 in the senescent cells compared with the young cells. All data are expressed as the mean ± standard error of mean (SEM). ^*^ *p* < 0.05; ^**^ *p* < 0.01; ^***^ *p* < 0.001.

This study aimed to identify the phenotype of senescent endothelial cells by examining various senescence‐related markers in human umbilical vein endothelial cells (HUVECs). The proportion of cells exhibiting positive β‐galactosidase staining was greater in replicative senescent cells compared with young cells (Figure 1A). Additionally, the mRNA and protein levels of senescence‐related markers p16, p21, and p53 were higher in the replicative senescent cells than in the young cells (Figure 1B; Figure S1). In comparison to young cells, replicative senescent cells exhibited elevated levels of several genes associated with inflammatory cytokines, cardiovascular function, and epigenetic regulation (Figure S1). However, the expression levels of certain critical genes, such as nitric oxide synthase 3, superoxide dismutase 2, sirtuin 3, and lysine methyltransferase 2D, were reduced (Figure S1). Dot blot analysis demonstrated a significant increase in the density of 5‐hmC in replicative senescent cells compared with young cells (Figure 1C). Immunofluorescence analysis further revealed that replicative senescent cells exhibited higher intensities of 5‐hmC and phosphorylated H2A.X variant histone (p‐γH2AX), a marker of DNA damage, relative to young cells (Figure 1D). To further investigate the alterations in 5‐hmC within senescent cells, PQ was employed to induce senescence in HUVECs. Following treatment with 20 µM PQ for 3 and 4 days, there was a significant upregulation in the mRNA levels of p16, p21, and p53 (Figure S2A). Additionally, both the number of cells positively stained for β‐galactosidase and the protein levels of p16, p21, and p53 were elevated after 3 days of 20 µM PQ treatment (Figure S2B,C). In addition, PQ also significantly induced senescence in H9C2 cells, human embryonic kidney (HEK) 293T cells, human kidney‐2 (HK2) cells, and human aortic vascular smooth muscle cells (T/G HA) cells (Figure S2D–G). Furthermore, cell cycle analysis revealed that PQ markedly increased the proportion of cells in the G2/M phase while decreasing the proportion of cells in the S phase (Figure S2H). These findings indicate that PQ is a potent inducer of cellular senescence.

To investigate the alterations of 5‐hmC in PQ‐induced aging mice, the animals were administered PQ intraperitoneally at a dosage of 10 mg/kg per week for 4 weeks to induce aging. The results demonstrated significant upregulation of mRNA levels of senescence‐associated markers p16, p21, and p53 in the heart, kidney, and aorta of PQ‐treated mice (Figure S3A–E). PQ treatment resulted in increased mRNA expression of inflammatory cytokines in the heart (Figure S3F,G), and induced myocardial fibrosis (Figure S3H). Additionally, levels of phosphorylated p‐γH2AX and 5‐hmC were significantly elevated in the heart, kidney, and aorta of PQ‐treated mice (Figure 1E,F; Figure S3I). However, PQ treatment did not induce apoptosis in the heart and kidney tissues of PQ‐induced aging mice (Figure S3J). These results indicated that 5‐hmC levels were markedly elevated in senescent cells and cardiovascular tissues of PQ‐induced aging mice.

To explore the alterations in DNA methylcytosine (5‐mC) and 5‐hmC levels in replicative senescent HUVECs, methylated DNA immunoprecipitation sequencing (MeDIP‐seq) and hydromethylated DNA immunoprecipitation sequencing (hMeDIP‐seq) were investigated. Bioinformatic analysis of the sequencing data revealed significant changes in DNA 5‐hmC levels compared with 5‐mC levels between the replicative senescent cells and the young cells. Specifically, a greater increase in the levels of 5‐hmC was found in the replicative senescent cells than in the young cells (Figure S4A, Tables S1, S2). The levels of 5‐mC were relatively unchanged changed in p21, and p53, whereas the levels of 5‐hmC were substantially increased (Figure 1G,H; Figure S4B). Analysis of the gene ontology (GO) showed that at the biological phenotype level, 5‐hmC was involved in the induction of nitric oxide synthase activity, cholesterol catabolism, negative regulation of endothelial cell migration, histone lysine demethylation, and positive regulation of protein complex disassembly, whereas at the molecular function level, it was involved the activities of numerous enzymes, such as histone demethylase, and methyltransferase as well as in the activities of translation repressor, intracellular ligand‐gated calcium channel, and phospholipid‐translocating ATPase in replicative senescent endothelial cells (Figure S4C). These results indicated that replicative senescent endothelial cells had a higher susceptibility to changes in genomic 5‐hmC, but not 5mC.

### 2.2 TET3 controls the Cellular Senescence Phenotype

**FIGURE 2:**
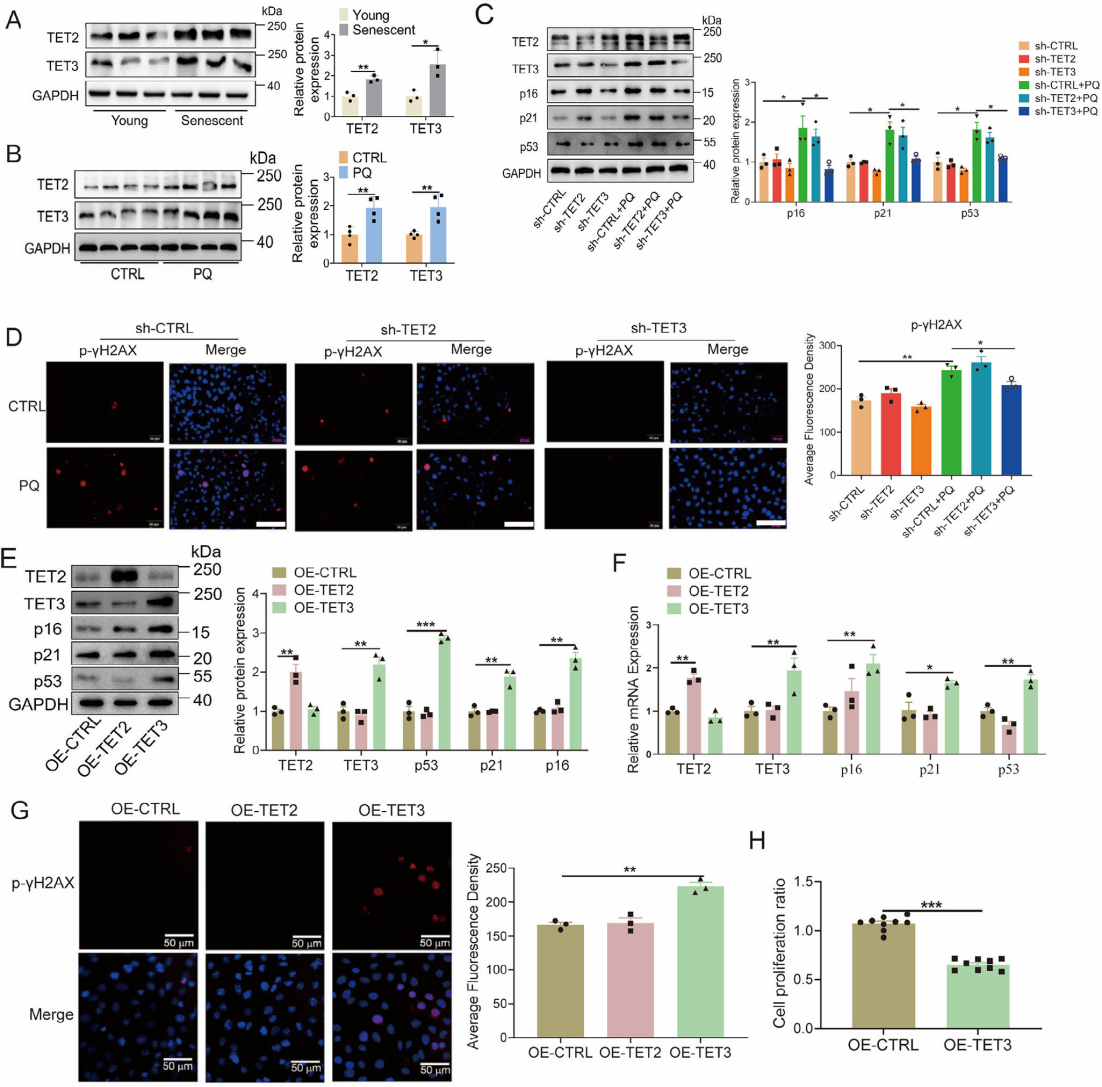
Role of TET3 in regulating cellular senescence. (A) Protein levels of TET2 and TET3 in replicative senescent HUVECs (*n* = 3 for each group, unpaired two‐sided *t*‐test). (B) Protein levels of TET2 and TET3 in HUVECs treated with PQ (*n* = 4 for each group, unpaired two‐sided *t*‐test). (C) Protein levels of p16, p21, and p53, and (D) Immunofluorescent imaging of p‐γH2AX after TET2 or TET3 silencing in HUVECs treated with PQ (*n* = 3 for each group, one‐way analysis of variance followed by Dunnett's multiple comparisons test, scale bars = 100 µm). (E, F) Protein and mRNA levels of p16, p21, and p53 after TET2 or TET3 overexpression (*n* = 3 for each group, unpaired two‐sided *t*‐test). (G) Immunofluorescent imaging of p‐γH2AX in HUVECs after TET2 or TET3 overexpression (*n* = 3 for each group, unpaired two‐sided *t*‐test, scale bars = 50 µm). (H) Cell proliferation after TET3 overexpression in HUVECs (*n* = 9 for each group, unpaired two‐sided *t*‐test). All the data are expressed as the mean ± SEM. ^*^ *p* < 0.05; ^**^ *p* < 0.01; ^***^ *p* < 0.001.

While some studies have suggested that the levels of TET2, and TET3 fluctuate with aging, either increasing or decreasing, definitive genetic evidence remains lacking [15, 33, 34]. This study aimed to elucidate the role of TET2 and TET3 in cellular senescence by examining their levels in senescent endothelial. The mRNA and protein levels of TET2 and TET3 were higher in the replicative senescent HUVECs compared with the young HUVECs, as well as in the PQ group compared with the control group (Figure 2A,B; Figure S5A,B). In addition, PQ treatment markedly increased the mRNA levels of TET2 and TET3 in HEK293T, and HK2 cells, and also markedly increased the mRNA level of TET3 in H9C2, and T/G HA cells (Figure S5C–F). Previous research has demonstrated that STAT3 can act as a transcription factor to regulate the expression of TET3 [35]. To investigate whether PQ modulates TET3 expression via STAT3, Western blotting and chromatin immunoprecipitation (ChIP) assays were conducted. The results revealed that PQ significantly enhances the phosphorylation of STAT3 and its binding to the TET3 promoter (Figure S5G,H). The knockdown of TET3, as opposed to TET2, effectively inhibited the upregulation of both protein and mRNA levels of p16, p21, and p53 in PQ‐induced senescent endothelial cells (Figure 2C; Figure S5I). Similarly, silencing TET3, but not TET2, inhibited the PQ‐induced enhancement in p‐γH2AX intensity within endothelial cells (Figure 2D). Moreover, overexpression of TET3, in contrast to TET2, resulted in elevated levels of p16, p21, and p53 in HUVECs (Figure 2E,F). This overexpression of TET3 also led to an increased intensity of p‐γH2AX in endothelial cells (Figure 2G) and significantly reduced proliferation of endothelial cells (Figure 2H).

**FIGURE 3:**
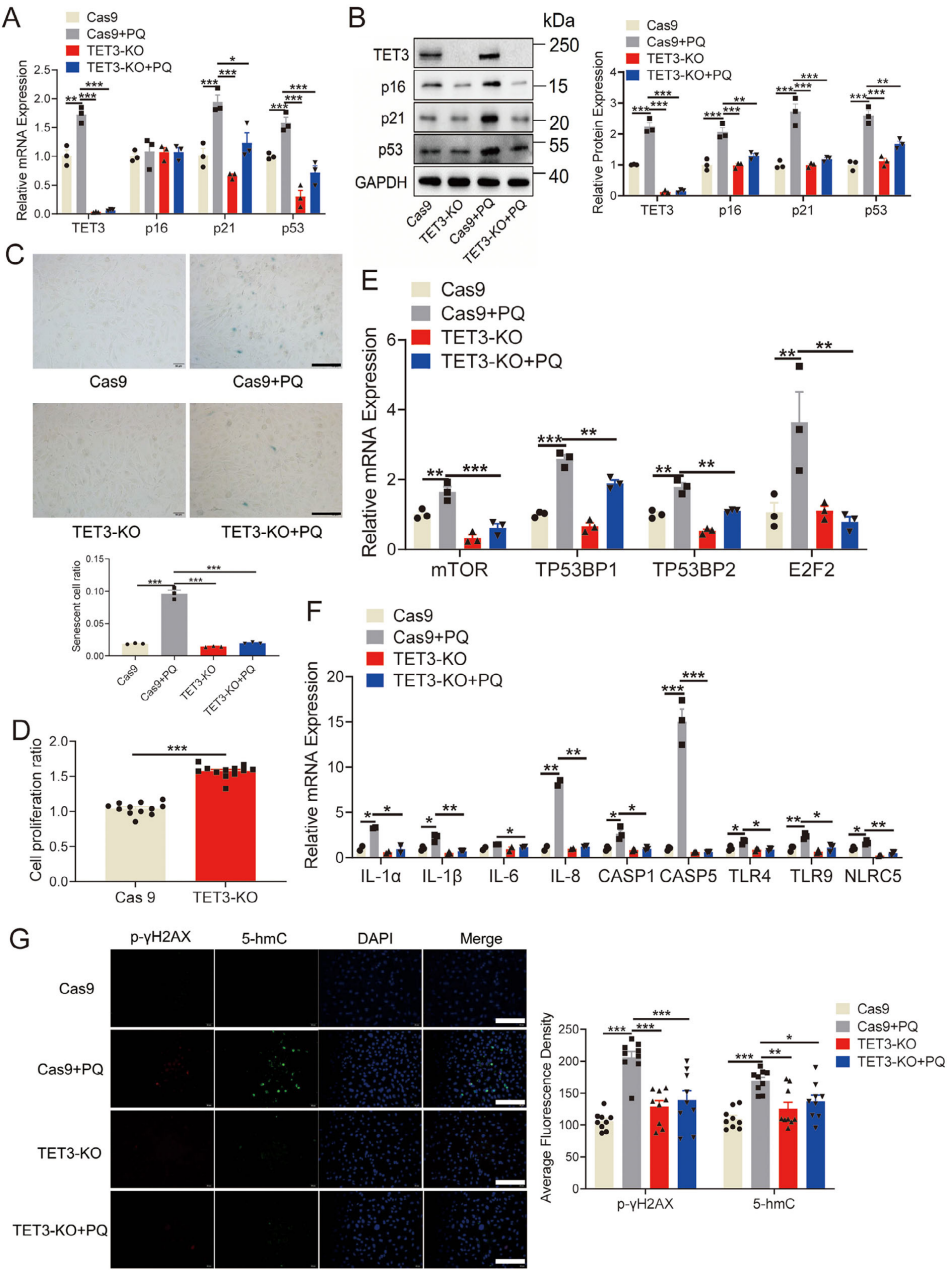
Role of TET3 knockout in regulating cellular senescence. (A, B) Protein and mRNA levels of p16, p21, and p53 in HUVECs with TET3 knockout (*n* = 3 for each group, one‐way analysis of variance followed by Dunnett's multiple comparisons test). (C) β‐galactosidase staining and senescent cell ratio in HUVECs with TET3 knockout (*n* = 3 for each group, one‐way analysis of variance followed by Dunnett's multiple comparisons test, scale bars = 100 µm). (D) Cell proliferation ratio in HUVECs with TET3 knockout (*n* = 12 for each group, unpaired two‐sided *t*‐test). (E, F) MRNA levels of SASP in HUVECs with TET3 knockout (*n* = 3 for each group, one‐way analysis of variance followed by Dunnett's multiple comparisons test). (G) Immunofluorescence imaging of p‐γH2AX and 5‐hmC in HUVECs with TET3 knockout (*n* = 9 for each group, one‐way analysis of variance followed by Dunnett's multiple comparisons test, scale bars = 100 µm). All data are expressed as the mean ± SEM. ^*^ *p* < 0.05; ^**^ *p* < 0.01; ^***^ *p* < 0.001.

To substantiate the role of TET3 in cellular senescence, the CRISPR/Cas9 technique was employed to delete the TET3 gene from HUVECs. The deletion of TET3 markedly reduced the PQ‐induced mRNA and protein levels of p16, p21, and p53 in HUVECs (Figure 3A,B). Additionally, TET3 deletion resulted in a decreased number of senescent HUVECs, as indicated by reduced β‐galactosidase staining (Figure 3C), and promoted cell proliferation in HUVECs (Figure 3D). TET3 deletion also significantly reduced the PQ‐induced mRNA levels of inflammatory cytokines (Figure 3E,F). Furthermore, the deletion of TET3 markedly suppressed the PQ‐induced elevation in the levels of p‐γH2AX and 5‐hmC in endothelial cells (Figure 3G). These findings indicate a potential role for TET3 in cellular senescence.

**FIGURE 4:**
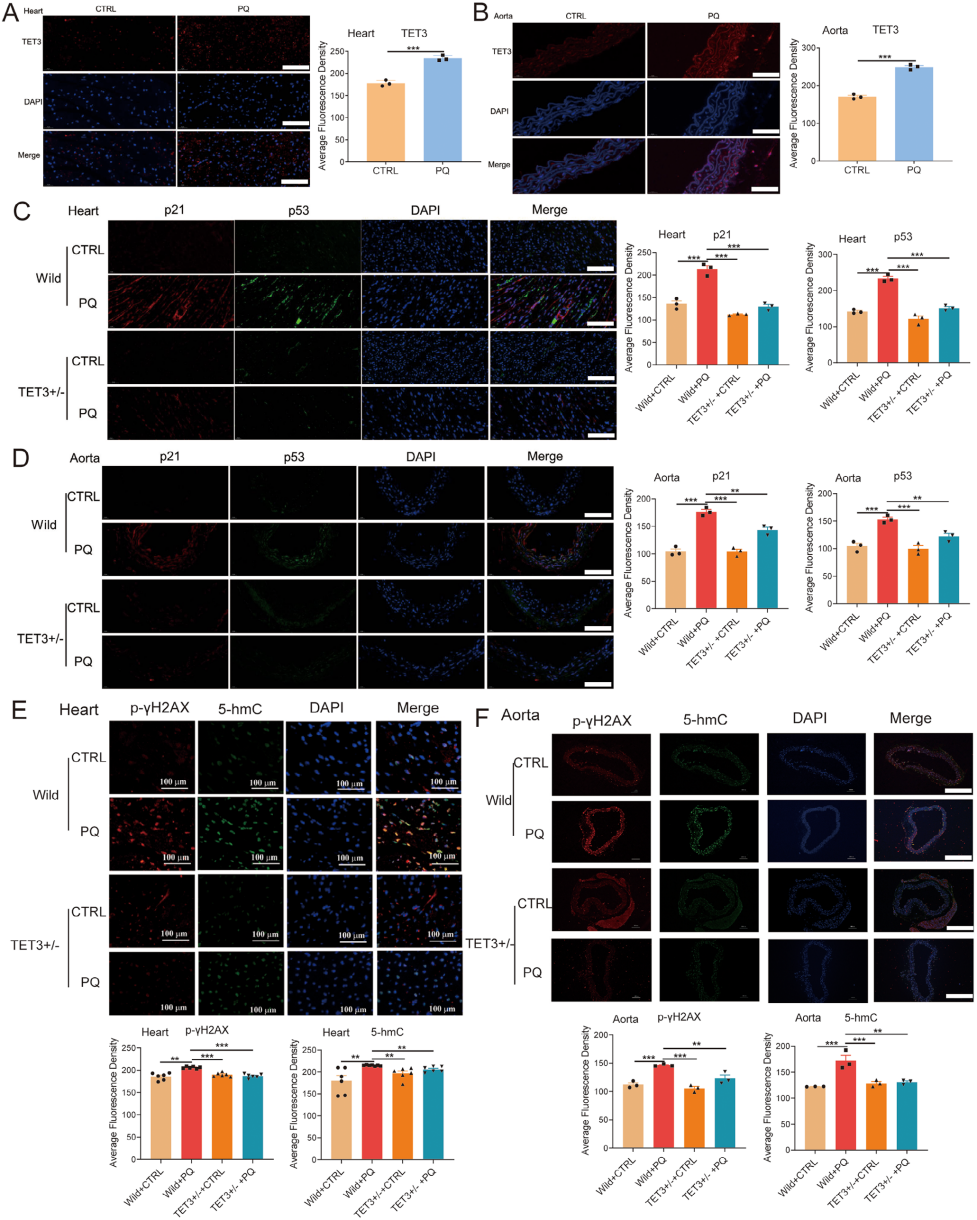
Role of TET3 in regulating aging in mice. (A, B) Protein level of TET3 in the heart and aorta tissues of PQ‐treated mice (*n* = 3 for each group, unpaired two‐sided *t*‐test, scale bars = 100 µm). (C, D) Protein levels of p21 and p53 in the senescent heart and aorta tissues of TET3‐heterozygous mice treated with PQ (*n* = 3 for each group, one‐way analysis of variance followed by Dunnett's multiple comparisons test, scale bars = 100 µm). Levels of p‐γH2AX and 5‐hmC in the senescent heart (E, scale bars = 100 µm) and aorta tissues (F, scale bars = 400 µm) of TET3‐heterozygous mice treated with PQ (*n* = 6 for E, and 3 for F each group, one‐way analysis of variance followed by Dunnett's multiple comparisons test). All data are expressed as the mean ± SEM. ^*^ *p* < 0.05; ^**^ *p* < 0.01; ^***^ *p* < 0.001.

Notably, TET3 expression was significantly upregulated in the heart, aorta, and kidney of mice treated with PQ (Figure 4A‐B, and Figure S6A–C). Given that homozygosity for TET3 results in neonatal sublethality [36], to further investigate the influence of TET3 on PQ‐induced aging mice, heterozygous TET3 mice were generated (Figure S6D). The results revealed that TET3 deficiency led to the inhibition of p21 and p53 in the heart and aorta of PQ‐induced aging mice (Figure 4C,D; Figure S6E). Moreover, TET3 deficiency led to a reduction in β‐galactosidase levels in the heart and aorta of PQ‐induced aging mice (Figure S6F,G), as well as decreased levels of p‐γH2AX and 5‐hmC in the aorta, heart, and kidney following PQ treatment (Figure 4E,F; Figure S6H). These results reinforce the role of TET3 as a key driver of the cardiovascular senescence.

### 2.3 TET3 Upregulates p53 Expression Through the 5‐hmC‐Mediated SP1‐ETS‐1 Axis

**FIGURE 5:**
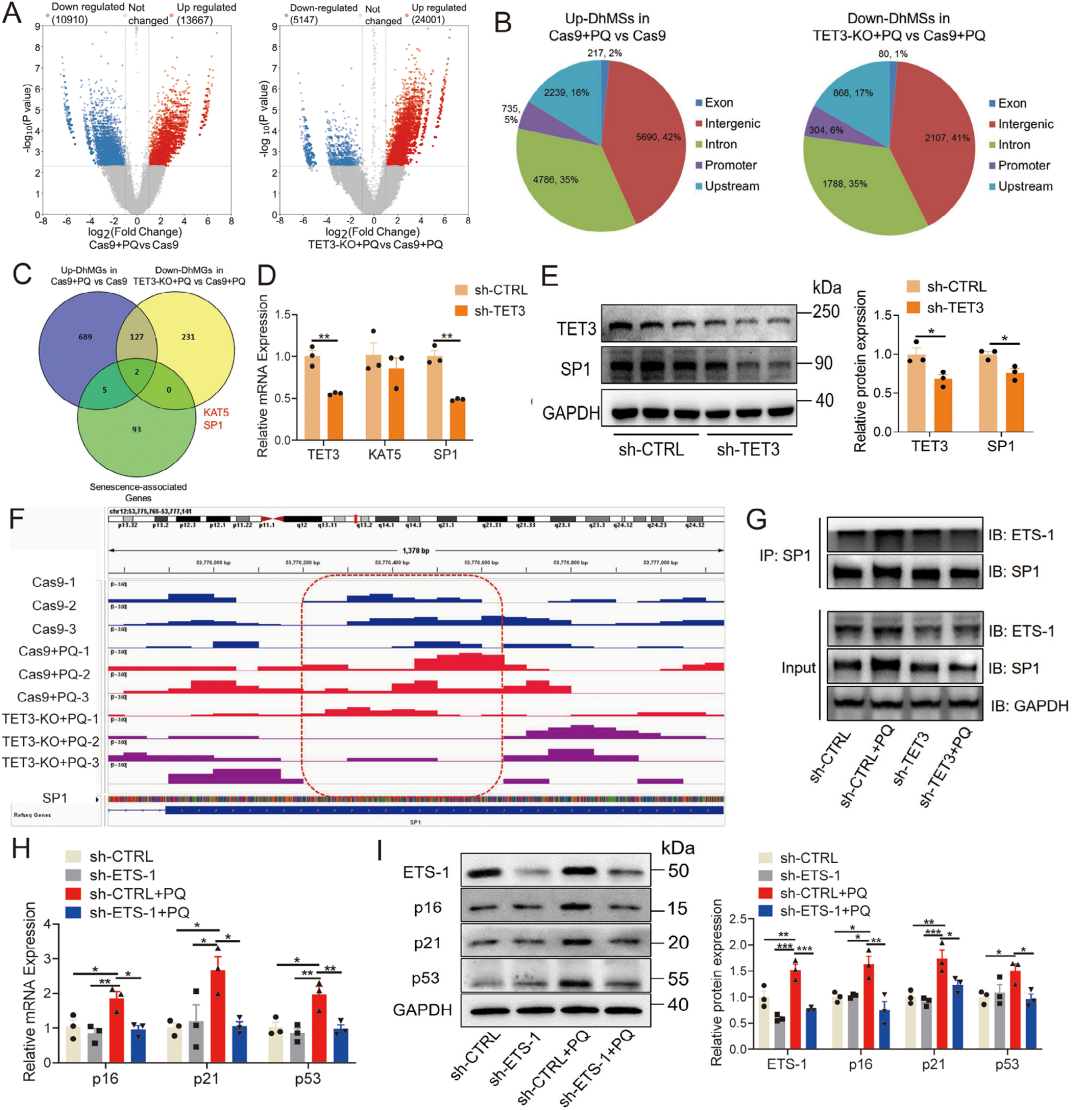
Regulation of TET3 on SP1‐ETS‐1 axis. (A) Changes in the total distribution of 5‐hmC in the genome in TET3 knockout/PQ‐treated HUVECs based on hMeDIP‐seq. (B) Distribution of DhMSs. (C) Overlapping DhMGs associated with senescence are shown using a Venn diagram. (D) Levels of KAT5 and SP1 mRNA after TET3 knockdown (*n* = 3 for each group, unpaired two‐sided *t*‐test). (E) Level of SP1 protein after TET3 knockdown (*n* = 3 for each group, unpaired two‐sided *t*‐test). (F) Level of 5‐hmC in SP1 protomer after TET3 knockdown. (G) The combination of SP1 and ETS‐1 after TET3 knockdown. (H, I) Protein and mRNA levels of p16, p21, and p53 after ETS‐1 knockdown (*n* = 3 for each group, one‐way analysis of variance followed by Dunnett's multiple comparisons test). All data are expressed as the mean ± SEM. ^*^ *p* < 0.05; ^**^ *p* < 0.01; ^***^ *p* < 0.001.

To elucidate the potential mechanism of TET3 in cardiovascular senescence, hMeDIP‐seq was conducted in HUVECs with TET3 deletion. The analysis indicated that the levels of 5‐hmC were predominantly localized at the transcriptional start site (Figure S7A). Importantly, despite the deletion of TET3, the distribution of 5‐hmC across various genomic regions, including intergenic, intronic, upstream, promoter, and exonic regions, remained largely unchanged (Figure S7B). Subsequently, the focus was directed toward differentially hydroxymethylated sites (DhMSs) that were upregulated in PQ‐induced HUVECs and downregulated following TET3 deletion. A total of 13,667 upregulated DhMSs were identified in PQ‐induced HUVECs, while 5147 downregulated DhMSs were identified in HUVECs with TET3 deletion, each exhibiting a fold change of >2, *p* < 0.005, and false discovery rate < 0.05 (Figure 5A; Figure S7C, Table S3). Furthermore, the proportion of DhMSs across different genomic regions (intergenic, intron, upstream, promoter, and exon) remained nearly consistent despite the deletion of TET3 (Figure 5B).

The GO and Kyoto Encyclopedia of Genes and Genome (KEGG) analyses revealed the involvement of different hydroxymethylated genes (DhMGs) in the regulation of aging, inflammation, and cancer. Specifically, PQ stimulation enhanced various signaling pathways related to viral infections, inflammatory responses, and multiple cancers (Figure S7D‐E). Knockout of TET3 inhibited the renin–angiotensin pathway, aldosterone synthesis and secretion, mitochondrial autophagy, and longevity‐regulating pathway. It downregulated the inflammatory response to leukocyte migration, RAS signaling transduction, and telomere DNA‐binding (Figure S7F,G). Furthermore, 5‐hmC may regulate gene expression by facilitating modifications of promoter CpG sites [37, 38]. In our study, we conducted an analysis of DhMGs in gene promoters, selecting those with a fold change greater than 2, a *p*‐value less than 0.005, and a false discovery rate below 0.05. Using a Venn diagram, we identified two overlapping DhMGs associated with senescence, namely SP1 and lysine acetyltransferase 5 (KAT5) (Figure 5C; Table S4). Although the levels of both SP1 and KAT5 were significantly increased in PQ‐induced senescent cells (Figure S8A,B), TET3 knockdown only significantly inhibited SP1 expression, not KAT5 (Figure 5D,E). Moreover, the 5‐hmC level of the SP1 promoter was significantly increased, and TET3 knockout significantly reduced its level (Figure 5F). These results indicated that TET3 may regulate SP1 expression in a 5‐hmC‐dependent manner.

Previous studies have demonstrated that SP1 can regulate transcription through cooperative interactions with ETS‐1 [39, 40, 41, 42, 43], which has been associated with human aging and the levels of senescence‐associated proteins [44, 45]. Therefore, the level and function of ETS‐1 were verified in cardiovascular senescence, and results revealed that mRNA and protein levels of ETS‐1 were significantly increased in the replicative senescent and the PQ‐induced senescent HUVECs (Figure S8C–F). The combination of SP1 and ETS‐1 was significantly increased in PQ‐induced senescent cells, whereas TET3 knockdown significantly reduced this combination and the level of ETS‐1 (Figure 5G). Moreover, ETS‐1 knockdown significantly reduced the levels of p16, p21, and p53 in PQ‐induced senescent cells (Figure 5H,I).

**FIGURE 6:**
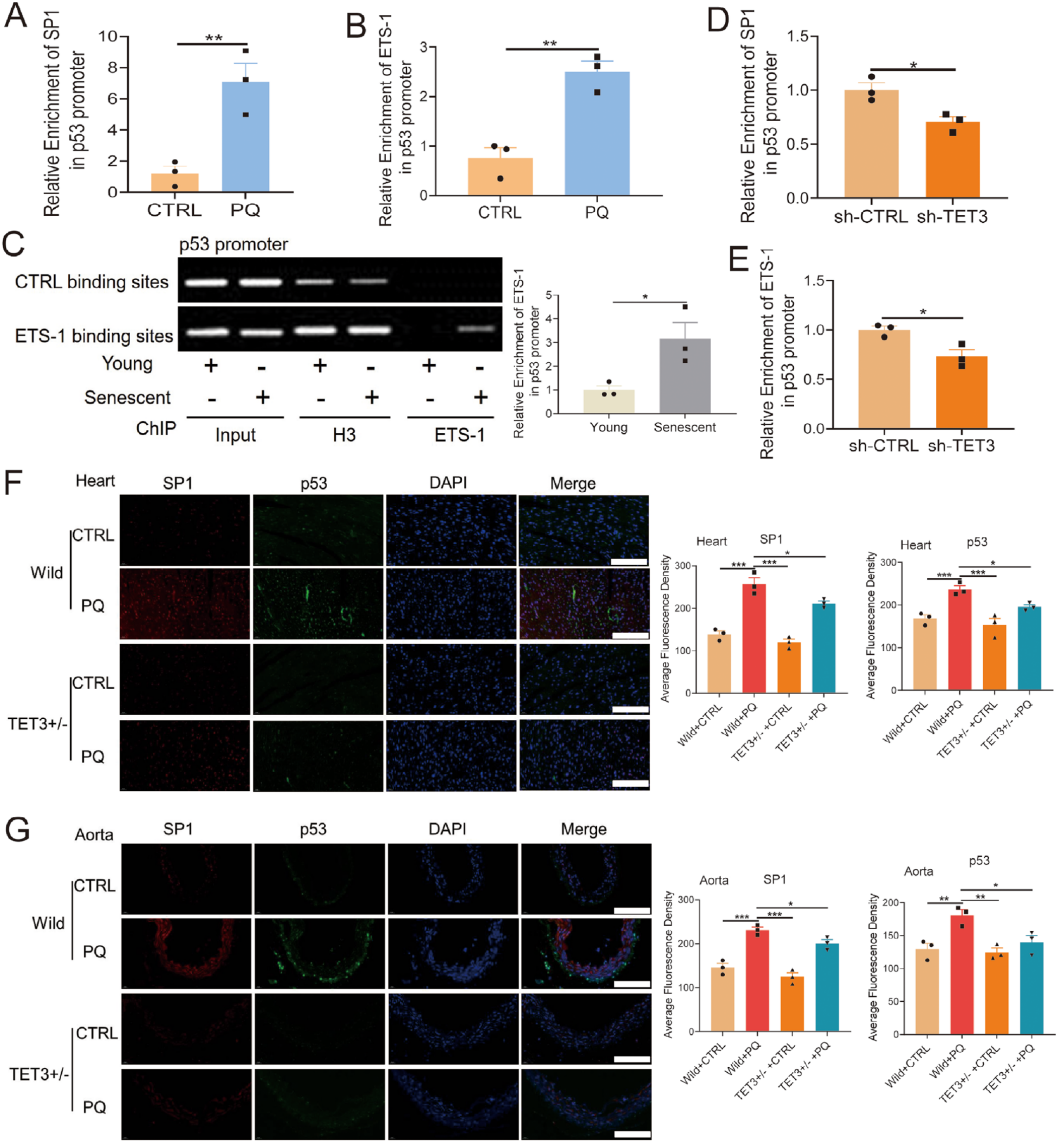
Role of SP1 and EST1 on p53 expression. (A) SP1 binding enrichment on the p53 promoter (*n* = 3 for each group, unpaired two‐sided *t*‐test). EST1 binding enrichment on the p53 promoter in PQ‐induced (B) or replicative (C) senescent HUVECs detected using ChIP assay (*n* = 3 for each group, unpaired two‐sided *t*‐test). (D, E) Binding enrichment of SP1 and ETS‐1 on the p53 promoter after TET3 knockdown (*n* = 3 for each group, unpaired two‐sided *t*‐test). (F, G) Levels of SP1 and p53 in the senescent heart and aorta tissues of TET3‐heterozygous mice treated with PQ (*n* = 3 for each group, one‐way analysis of variance followed by Dunnett's multiple comparisons test, scale bars = 100 µm). All data are expressed as the mean ± SEM. ^*^ *p* < 0.05; ^**^ *p* < 0.01.

ETS‐1 is known to induce cellular senescence by upregulating p53 expression [46, 47]. In this study, both TET3 knockdown and deletion significantly inhibited p53 expression (Figures 2, 3), whereas TET3 overexpression led to a marked increase in p53 expression (Figure 2E,F). These observations led us to hypothesize that TET3 modulates p53 expression via its interaction with the SP1‐ETS‐1 axis. To evaluate this hypothesis, we conducted a ChIP assay, which demonstrated a significant increase in SP1 enrichment at the p53 promoter in PQ‐induced senescent cells (Figure 6A). Additionally, the enrichment of ETS‐1 at the p53 promoter was also significantly increased in the replicative senescent and the PQ‐induced senescent HUVECs (Figure 6B,C). TET3 knockdown notably diminished the enrichment of SP1 and ETS‐1 at the p53 promoter (Figure 6D,E). Additionally, our findings indicated that TET3 deficiency reduced the levels of SP1 and p53 in the heart and aorta of aging mice induced by PQ (Figure 6F,G). These results suggested that TET3 regulated the expression of p53 by mediating the SP1‐ETS‐1 axis.

### 2.4 Interaction Between p53 and TET3 in Controlling Cellular Senescence

**FIGURE 7:**
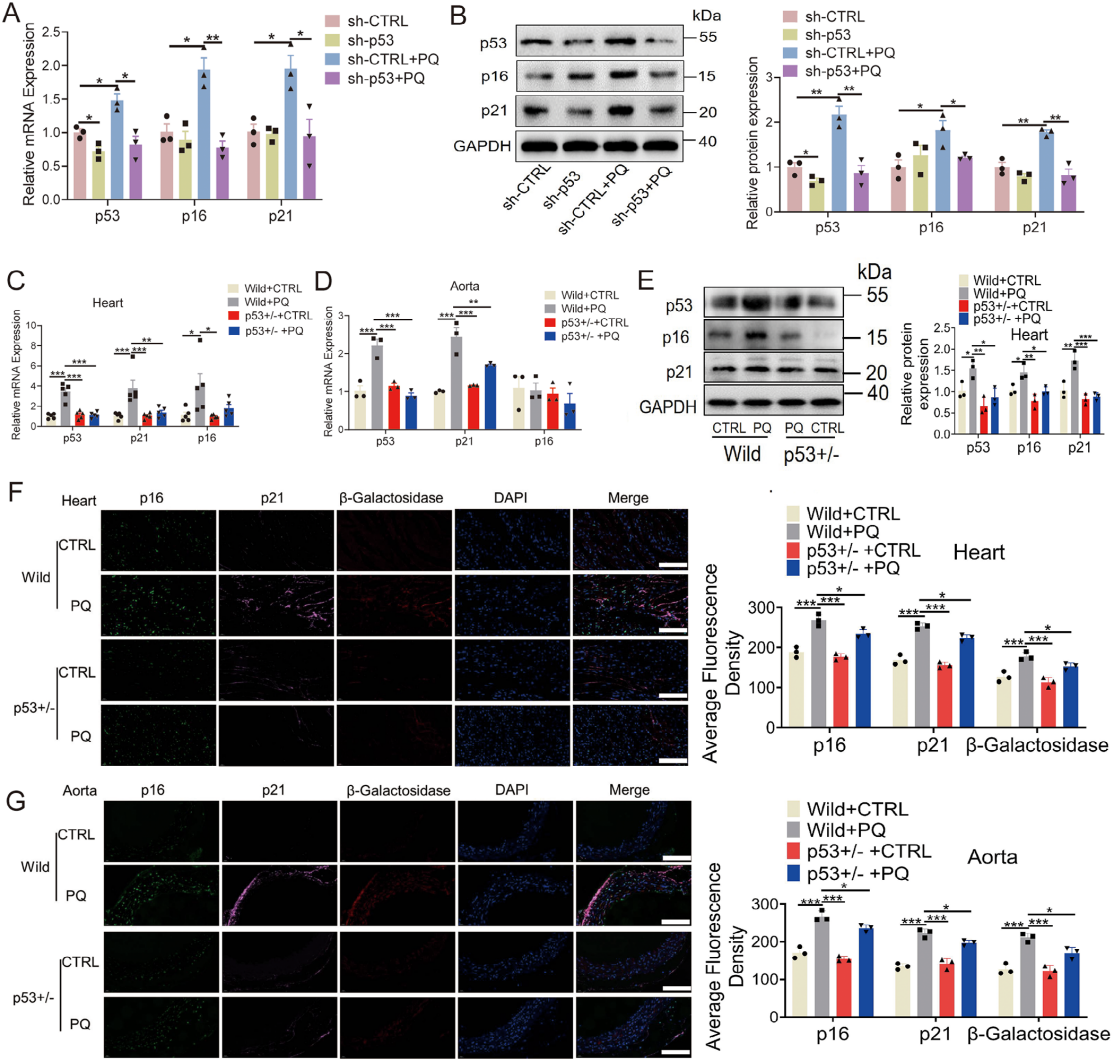
Role of p53 in regulating senescence in vivo and vitro. (A, B) Protein and mRNA levels of p16, p21, and p53 after p53 knockdown (*n* = 3 for each group, one‐way analysis of variance followed by Dunnett's multiple comparisons test). (C, D) Expression of p53, p16, and p21 mRNAs in the heart and aorta tissues of p53^+^/^−^‐ heterozygous mice treated with PQ (*n* = 5 for C, and 3 for D each group, one‐way analysis of variance followed by Dunnett's multiple comparisons test). (E) Protein levels of p53, p16, and p21 in the heart tissues of p53^+^/^−^‐heterozygous mice treated with PQ (*n* = 3 for each group, one‐way analysis of variance followed by Dunnett's multiple comparisons test). (F, G) Levels of p16, p21, and β‐galactosidase in the heart and aorta tissues of p53^+^/^−^‐heterozygous mice treated with PQ (*n* = 3 for each group, one‐way analysis of variance followed by Dunnett's multiple comparisons test, scale bars = 100 µm). All data are expressed as the mean ± SEM. ^*^ *p* < 0.05; ^**^ *p* < 0.01; ^***^ *p* < 0.001.

The role of p53 in senescence and aging has been well established [48]. In the present study, the knockdown of p53 resulted in decreased levels of p16 and p21 (Figure 7A,B). To further elucidate the impact of p53 on aging in vivo, p53‐heterozygous mice were generated (Figure S9A). Our findings demonstrated that mRNA levels of p21 were significantly reduced in the hearts, kidneys, and aortas of p53^+/−^‐heterozygous mice induced by PQ treatment (Figure 7C,D; Figure S9B,C). Moreover, the protein levels of p16, p21, and β‐galactosidase were also markedly reduced in the hearts and aortas of p53^+/−^‐heterozygous mice (Figure 7E–G).

**FIGURE 8:**
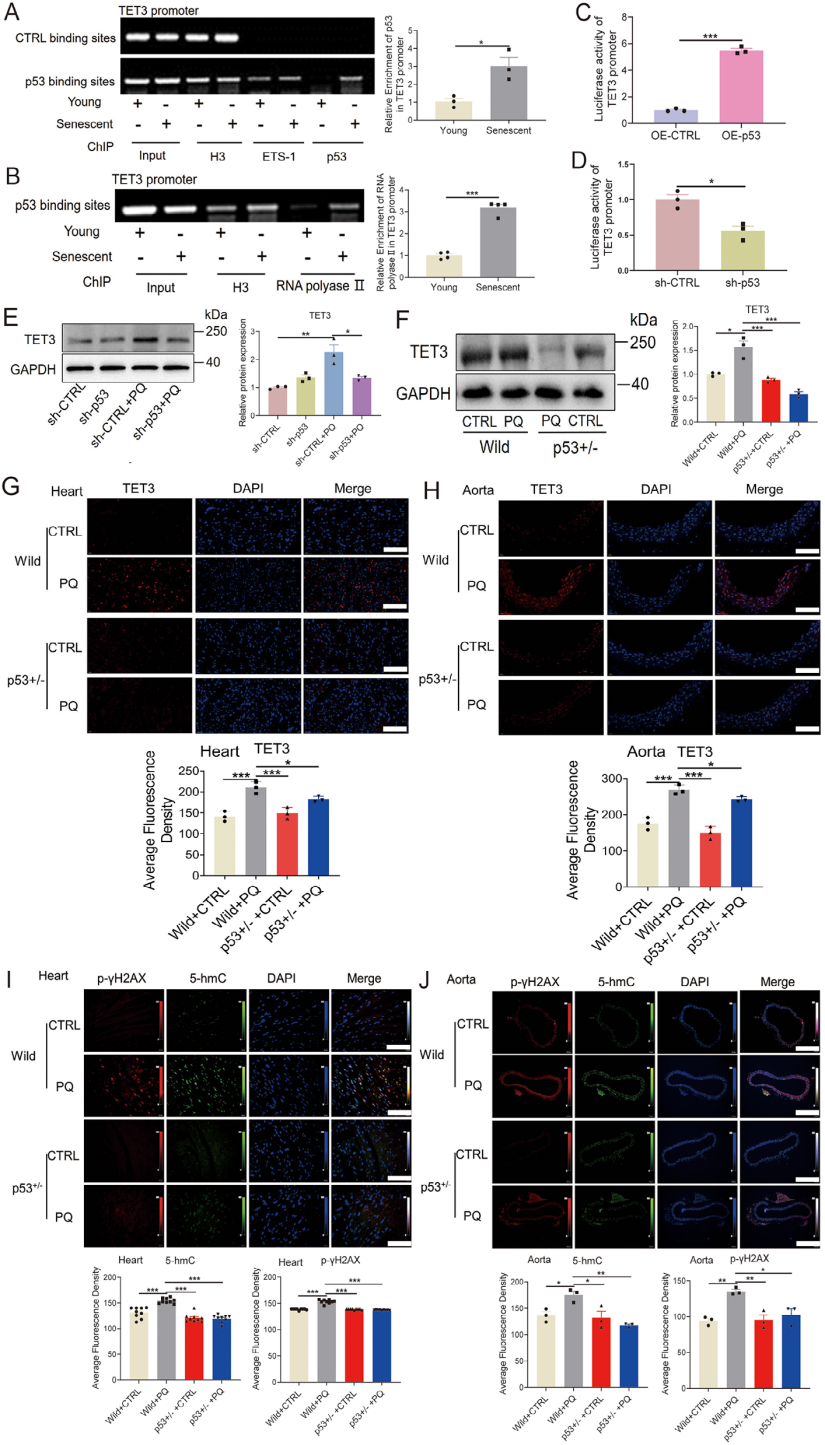
Regulation of TET3 expression by p53. The capacity of the p53 protein (A) or RNA polymerase II (B) to bind to the TET3 promoter, determined using the ChIP assay in HUVECs (*n* = 3 for each group, unpaired two‐sided *t*‐test). (C, D) Luciferase reporter analysis of TET3 activity after p53 overexpression or silencing (*n* = 3 for each group, unpaired two‐sided *t*‐test). (E) Protein level of TET3 after p53 knockdown. (F) Protein level of TET3 in the heart tissues of p53^+^/^−^‐heterozygous mice treated with PQ (*n* = 3 for each group, one‐way analysis of variance followed by Dunnett's multiple comparisons test). (G, H) Levels of TET3 in the heart and aorta tissues of p53^+^/^−^‐heterozygous mice treated with PQ (n = 3 for each group, one‐way analysis of variance followed by Dunnett's multiple comparisons test, scale bars = 100 µm). Levels of p‐γH2AX and 5‐hmc in the heart (I, scale bars = 100 µm) and aorta tissues (J, scale bars = 400 µm) of p53^+^/^−^‐heterozygous mice treated with PQ (*n* = 9 for I, and 3 for J each group, one‐way analysis of variance followed by Dunnett's multiple comparisons test). All data are expressed as the mean ± SEM. ^*^ *p* < 0.05; ^**^ *p* < 0.01; ^***^ *p* < 0.001.

TET3 affected the expression of p53 through the 5‐hmC‐mediated SP1‐ETS‐1 axis. Further analysis revealed a binding element of p53 at the proximal end of the TET3 promoter. To investigate whether p53 can also negatively regulate TET3 expression [49], a ChIP assay was performed, revealing that p53 binds to the TET3 promoter (Figure 8A). Furthermore, RNA polymerase II was also observed to bind to the p53 binding site on the TET3 promoter (Figure 8B). In addition, the results of the luciferase reporter assay for the TET3 promoter revealed that p53 overexpression or silencing significantly activated or prevented TET3 promoter activity (Figure 8C,D; Figure S10A,B). P53 knockdown also significantly reduced the levels of TET3 in PQ‐induced senescent cells (Figure 8E; Figure S10C). The levels of TET3 were decreased in the hearts, kidneys, and aortas of p53^+/−^‐heterozygous mice induced by PQ (Figure 8F–H; Figure S10D–E). Furthermore, the levels of p‐γH2AX and 5‐hmC were significantly decreased in the aortas, hearts, and kidneys of p53^+/−^‐heterozygous mice that received PQ treatment (Figure 8I,J; Figure S9D). These data revealed the interdependence of p53 and TET3 in controlling cardiovascular senescence.

## 3 Discussion

5‐hmC is recognized as being a stable epigenetic modification [50], capable of binding specific proteins [51, 52], such as ubiquitin‐like with PHD and ring finger domains 2 (Uhrf2), which act as 5‐hmC readers in neuronal progenitor cells, with strong specificity [52]. The specific binding of Uhrf2 to 5‐hmC has been shown to be dynamic during cell differentiation, indicating that 5‐hmC functions as an epigenetic signal with specific biological effects [52]. Moreover, many studies have demonstrated that 5‐hmC is enriched in specific regulatory regions, such as the promoter [37, 38], where it suppresses their activity [53, 54]. In this study, levels of 5‐hmC were markedly elevated in replicative senescent HUVECs, PQ‐induced HUVECs, and aging mice. The distribution of DhMSs was more pronounced on promoters in the replicative senescent HUVECs, indicating that 5‐hmC may regulate gene expression by mediating CpG modifications of gene promoters, consistent with previous findings [37, 38]. Moreover, deletion of TET3 resulted in a decreased 5‐hmC level on the SP1 promoter, leading to reduced SP1 expression and subsequent regulation of cellular senescence. Similar studies have demonstrated that TET1 overexpression led to an increase in SP1 expression as well as the upregulation of p53 in spermatogonia [55]. Therefore, this loop formed by TET1/3‐5‐hmC‐SP1 is a novel mechanism that drives p53 expression and cellular senescence. Thus, TET3‐mediated 5‐hmC, as a stable epigenetic signaling molecule, plays a vital role in cardiovascular senescence.

The subtypes of the TET family exhibit distinct functions; however, the specific role of TET3 in disease and cellular senescence remains to be elucidated. Although several studies have demonstrated that aging is associated with changes in the hydroxymethylation of brain tissue and blood cells, which TET subtype regulates this process remains inconclusive. In this study, TET3 was identified as a pivotal active DNA demethylase with a significant role in facilitating cellular senescence. The expression of TET3 was significantly elevated in senescent endothelial cells and cardiovascular tissues, further supporting its involvement in aging‐related processes. Overexpression of TET3, as opposed to TET2, enhanced endothelial cellular senescence, whereas silencing or deletion of TET3 inhibited cellular senescence and levels of proinflammatory cytokines. Moreover, TET3 deficiency delayed aging in mice treated with PQ. A previous study revealed that silencing of TET3 reduced cardiac fibrosis and hydroxymethylation induced by bone morphogenetic protein 7 [56]. In addition, senescence‐associated secretory phenotype (SASP) has been shown to contribute to senescence‐related inflammation and aging phenotypes [57, 58, 59]. In the current study, TET3 was shown to elevate SASP levels, whereas TET3 inhibition led to a reduction in SASP levels. These findings suggest that TET3 regulated aging phenotypes.

Prior studies have shown that the cooperative activation of SP1 and ETS‐1 can regulate gene expression [40–43, 60]. Moreover, ETS‐1 has been shown to enhance p16 expression [45] and promote cellular senescence by increasing the expression of ribosomal protein genes [44]. In our study, the levels of SP1 and ETS‐1 were significantly elevated in PQ‐induced senescent cells and aging mice. Furthermore, the deletion of TET3 led to a reduction in SP1 and ETS‐1 levels through the mediation of 5‐hmC modification. The combined expression of SP1 and ETS‐1 was significantly increased in PQ‐induced senescent cells, whereas TET3 knockdown significantly reduced this combination. Additionally, knockdown of ETS‐1 markedly reduced cellular senescence, corroborating previous findings [44, 47]. Prior studies have shown that ETS‐1 can induce cellular senescence by promoting the expression of p53 [46, 47]. The current study confirmed the regulatory role of ETS‐1 on p53 expression, demonstrating that both SP1 and ETS‐1 bind to the p53 promoter to enhance its expression and induce cellular senescence. Moreover, TET3 inhibited the binding of SP1 and ETS‐1 to the p53 promoter, suggesting that TET3 regulated the aging phenotypes by mediating the SP1–ETS‐1–p53 axis. Furthermore, one confirmed SP1 binding site and five putative SP1 binding sites were found on the ETS‐1 promoter [61, 62], suggesting that SP1 not only synergizes with ETS‐1 to regulate the gene expression but also promotes the increase of p53 by increasing ETS‐1 expression.

Despite extensive research on p53 biology, investigations into the relationship between p53 genes and 5‐hmC remain limited. Previous studies have indicated that the loss of genomic methylation is associated with p53‐dependent apoptosis and epigenetic disorders, suggesting a potential role for p53 in the regulation of DNA methylation [63]. Furthermore, research has shown that p53 inactivation and the reduced methylation can lead to the transcription of tandem repeats and multiple noncoding RNAs in p53‐deficient mouse fibroblasts, which are typically inhibited by the combination of p53 protein and DNA methylation [64]. In p53‐null mouse embryonic stem cells, there is a marked reduction in the expression of TET1 and TET2, while DNA methyltransferase 3A and DNA methyltransferase 3B were significantly upregulated [65]. These findings suggest that p53 facilitates the expression of TET1 and TET2. However, evidence of p53 directly regulating the expression and activity of the TET family is lacking. In the present study, p53 was identified as an activating transcription factor for TET3 expression, and the knockdown or overexpression of p53 inhibited or increased TET3 expression, respectively. Our results confirmed that p53 binds to the p53 element region of the TET3 promoter for regulating TET3 expression to induce cellular senescence. DNA damage triggers the p53‐dependent regulation of cellular senescence positioning cellular senescence as a protective mechanism against DNA damage. Our findings demonstrated a correlation between p‐γH2AX and TET3 expression. Furthermore, programmed cellular senescence during mammalian embryonic development serves as a normal mechanism for tissue remodeling [66, 67]. TET3 may also contribute to the regulation of programmed cellular senescence during embryonic development, as evidenced by changes in the 5‐hmC landscape of the p21 gene, rather than 5‐mC, in senescent cells [36]. The findings revealed that p53 acts as a regulator of TET3 expression, suggesting an interdependent relationship between p53 and TET3, at least in the context of cellular senescence. However, the study has still some limitations. First, this study has not yet pinpointed the specific regions within the genomic 5‐hmC landscape that are linked to age‐related diseases. Further investigations are required to validate these findings. Second, although the disruption of 5‐hmC signaling through TET3 knockdown attenuated the PQ‐induced upregulation of gene expression in pathways associated with cardiovascular disease, we could not individually resolve their 5‐hmC modifications.

### 3.1 Summary

This study demonstrated that TET3 played a critical role in regulating cardiovascular senescence. These findings offer the new perspective that TET3 plays a role in cardiovascular senescence. Targeting TET3 activity is a potential therapeutic strategy for age‐related cardiovascular diseases.

## 4 Materials and Methods

### 4.1 Experimental Animal Model

C57BL/6J male mice were purchased from Shanghai SIPPR‐BK Laboratory Animal Co. Ltd (Shanghai, China). TET3‐heterozygous and p53‐heterozygous mice were commercially obtained from the Shanghai Model Organisms Center, Inc. (Shanghai, China). Because the TET3 homozygote may lead to neonatal sublethality, we used TET3‐heterozygous mice for the experiments.

The mice were housed in a specific pathogen‐free animal facility. Six‐week‐old male mice, including C57BL/6J, TET3^+/−^ mice, and p53^+/−^ mice, were intraperitoneally injected with PQ (Yuanye Biotechnology, Shanghai) at a dose of 10 mg/kg per week or phosphate‐buffered saline (control). All the mice were euthanized after 4 weeks. Subsequently, the hearts, kidneys, and aortas were excised, weighed, and cryopreserved with liquid nitrogen, with a portion of the tissues sectioned for immunofluorescence.

### 4.2 Cell Line and Culture

HUVECs (CRL‐1730) and human vascular smooth muscle cells (T/G HA, CRL‐1999) were purchased from the American Type Culture Collection. HEK 293T cells, human kidney‐2 (HK‐2), and rat H9C2 cardiomyocytes were obtained from the Cell Bank of the Chinese Academy of Sciences.

HUVECs were cultured in Dulbecco's Modified Eagle Medium supplemented with 10% newborn calf serum. The H9C2 cardiomyocytes and HEK293T cells were maintained in Dulbecco's Modified Eagle Medium supplemented with 10% fetal bovine serum. T/G HA cells were cultured in F‐12K medium and HK‐2 cells were cultured in K‐SFM medium according to the manufacturer's protocol. The cell lines were cultured in a humid environment with 5% CO_2_ and 95% air at a temperature of 37°C.

### 4.3 Senescent Cell Model

Replicative senescence in HUVECs was induced by subculturing the cells to a population doubling level (PDL) of 50 through repeated divisions [3]. Young cells were subcultured to a PDL of 10. PDL refers to the total number of times the cells in the population have doubled since their primary isolation in vitro. The PDL was calculated as follows: PDL = 3.32 (log UCY − log l) + X, where n is the final PDL at the end of a given subculture, UCY is the cell yield at that point, l refers to the cell number used as inoculum to begin that subculture, and X denotes the doubling level of the inoculum used to initiate the quantitated subculture. The cumulative population doubling was calculated as the sum of all the changes in population doubling during the subculturing process. Subsequently, the HUVECs were harvested for senescent staining, immunofluorescence, immunoblot, and quantitative polymerase chain reaction (qPCR) analyses.

To establish a PQ‐induced senescent cell model, HUVECs were plated on 6‐well plates with a density of 5 × 10^5^ cells per well, and cultured with PQ (20 µM) for 2, 3, and 4 days. Cells were cultured in the complete medium and served as negative controls. The cell proliferation was conducted following the manufacturer's instructions, utilizing the cell counting kit‐8 (Beyotime Biotech, Nantong, China). Additionally, the staining of β‐galactosidase was performed to identify cellular senescence and the expression of senescence‐related genes was also measured. Moreover, the induction of cellular senescence by 20 µM PQ was tested in HK‐2, T/G HA, H9c2, and HEK293T cells after a prolonged incubation duration of 3 days. Furthermore, gene‐modified HUVECs of p53 or TET overexpression, knockout, and knockdown were treated with 20 µM PQ for 3 days.

### 4.4 MeDIP‐seq and hMeDIP‐seq

To investigate the specific genomic regions where the levels of 5mC and 5‐hmC were altered in senescent endothelial cells, MeDIP‐seq and hMeDIP‐seq were performed. The MeDIP‐seq and hMeDIP‐seq process included labeling, hybridization, scanning, normalization, and data analysis, which was conducted by KangChen Biotech Inc. (Shanghai, China) and CloudSeq Biotech Inc. (Shanghai, China).

### 4.5 TET3 knockout by Targeting the CRISPR/cas9 Gene

For TET3 knockout (KO) in HUVECs, the TET3 CRISPR/Cas9 KO plasmid(h) (sc‐414997) and TET3 HDR plasmid(h) (sc‐414997‐HDR) were used, with the control CRISPR/Cas9 plasmid (sc‐418922) as negative control, all of which were purchased from Santa Cruz. The HUVECs were seeded in a 24‐well plate at a density of 2 × 10^5^. Transfection of these plasmids was performed using FuGENE HD transfection reagent (Promega), and the cells were incubated for 6 h. After transfection, the cells were cultured in a fresh normal medium and subjected to puromycin selection (1 µg/mL) to enrich positive cells according to previous studies [68, 69]. After puromycin selection, the survival cells were plated on 96‐well plates at a single‐well density, and cell clones were screened using qPCR to confirm successful TET3 gene knockout.

### 4.6 shRNA Transfection

For gene silencing, 8 µg of shRNA plasmids (h) targeting TET2 (sc‐88934‐SH), TET3 (sc‐94636‐SH), p53 (sc‐29435‐SH), and ETS‐1 (sc‐29309) were individually transfected into HUVECs. Additionally, a shRNA plasmid‐control (sc‐108060) was used as a control for comparison. After 6 h of transfection, 20 µM PQ was added to the cell culture medium. Subsequently, after 3 days, the cells were harvested for functional experiments.

### 4.7 Transfection of Gene Overexpression

To overexpress human TET2, TET3, or p53 genes, their cDNA was inserted into the pCDH‐CMV‐MCS‐EF1‐Green plasmid (CD513B‐1). The cells were placed in a six‐well dish with a concentration of 5 × 10^5^ cells per well. Next, the cells were transfected with 8 µg of the TET2, TET3, or p53 plasmid or control plasmid by using the FuGENE HD transfection reagent (Promega) following the protocol established in previous studies [68, 69]. After 6 h of transfection, the transfection medium was replaced with a fresh normal medium, and the cells were incubated in a normal medium for an additional 24 h before protein or RNA extraction.

### 4.8 β‐Galactosidase Senescence Staining

The cellular senescence assay kit (Cell Biolabs, Inc.) was used to stain senescent cells after fixing them in 4% paraformaldehyde. Staining was allowed to occur overnight at 37°C. After staining, the staining solution was removed. After incubation for 24 h, photographs of the stained cells were taken using a standard light microscope (Olympus).

### 4.9 RNA Extraction and qPCR

Total RNA of snap‐frozen tissues or cultured cells was extracted by using TRIzol reagent (Thermo Fisher Scientific). The cDNA reverse transcription kit (Thermo Fisher Scientific) was utilized for cDNA synthesis as per the manufacturer's guidelines. QPCR analyses were performed. All primers (Table S5) used were obtained from Generay (Generay Biotech, Shanghai). Values were normalized to glyceraldehyde‐3‐phosphate dehydrogenase (GAPDH). Relative expression values were calculated using the 2^−ΔΔCt^ method, and the results are expressed as relative fold changes.

### 4.10 Western Blot Analysis

To perform western blot analysis, snap‐frozen tissues and cells were both homogenized in radioimmunoprecipitation assay lysis buffer (Beyotime Biotechnology, Nantong, China). After centrifugation, the lysates were quantified to prepare for gel electrophoresis. Samples were loaded and separated and then transferred onto NC membranes (HATF04700, Merck Millipore). Next, the membranes were blocked for 1 h at room temperature and incubated with primary antibodies overnight at 4°C. Primary antibodies against p16 (1:1000, ab108349, Abcam), p21 (1:250, sc‐397, Santa Cruz), p53 (1:1000, 2524, CST), TET2 (1:250, sc‐136926, Santa Cruz), TET3 (1:250, sc‐139186, Santa Cruz), SP1 (1:1000, 9389, CST), ETS‐1 (1:1000, 14069, CST), STAT3 (1:1000, 9139, CST), p‐STAT3 (1:1000, 9145, CST), β‐actin (1:5000, 1702–67, Huabio), and GAPDH (1:5000, KC‐5G5, KangChen Bio‐tech Inc., Shanghai, China) were used. Proteins were detected and quantified using ImageJ software.

### 4.11 Dot Blot

The extraction of genomic DNA was performed using the genomic DNA mini preparation kit (D0063, Beyotime Biotech). The DNA samples were subjected to denaturation at a temperature of 99°C for 5 min. Subsequently, the denatured samples were applied onto nitrocellulose membranes. The membranes were treated using ultraviolet for 30 min and blocked with 5% nonfat milk for 2 h, and incubated with 5‐hmC antibodies (1:500, ab106918, Abcam) overnight at 4°C. Then, HRP‐conjugated goat antimouse antibodies were applied and then captured. To track DNA loading, membranes were stained using a solution of 0.02% methylene blue in 0.3 M sodium acetate (with a pH of 5.2).

### 4.12 Immunofluorescence Staining

The heart, kidney, and aorta tissues were fixed with 4% paraformaldehyde overnight. The tissues were then dehydrated and embedded in paraffin for sectioning. Before staining, the samples were treated with 4% paraformaldehyde for 15 min, followed by permeabilization with 0.5% triton X‐100 for 15 min. Then the tissue sections or cells were incubated with primary antibodies overnight at 4°C, followed by incubation with secondary antibodies for 1 h. The primary antibodies used were against 5‐hmC antibody (1:200, 39769, Active Motif), p‐γH2AX antibody (1:200, ab26350, Abcam), SP1 (1:100, A19649, ABclonal), TET3 (1:200, GTX‐121453, GENETEX), β‐galactosidase (1:100, 27198, CST), p16 (1:100, sc‐1661, Santa Cruz), p21 (1:100, sc‐6246, Santa Cruz), and p53 (1:100, 2524, CST). The stained cells were observed and imaged using an Olympus IX71 fluorescence microscope (Olympus). Images were processed using the cellSens software (Olympus) and quantified using ImageJ software.

### 4.13 Luciferase Reporter Assay

HEK 293T cells were transfected with p53 shRNA plasmid (sc‐29435‐SH, Santa Cruz) or human p53 overexpression plasmid for 24 h. Then, the 293T cells were cotransfected with a mixture of the TET3 promoter plasmid and the pRL‐SV40 plasmid containing *renilla* luciferase gene. After 6 h of cotransfection, the culture medium was replaced, and the cells were cultured for 16 h. The luciferase assays were conducted utilizing the dual‐luciferase reporter assay kit (E1980, Promega) following previous studies [69, 70], and the luciferase activity was measured using a Varioskan Flash microplate spectrophotometer (Thermo Scientific).

### 4.14 Immunoprecipitation Assay

The immunoprecipitation assay was performed using an assay kit (YJ003, EpiZyme, China) according to a previous study [71]. Briefly, the cells were prepared and homogenized for 10 min. The lysates were then collected through centrifugation to remove cell debris. The samples were incubated with protein A/G magnetic beads and SP1 (9389, CST) antibody overnight at 4°C. Finally, the samples were washed and denatured for subsequent western blotting analysis.

### 4.15 Chromatin Immunoprecipitation Assay

The ChIP assay was performed using an assay kit (ab500, Abcam). Briefly, the cells were crosslinked with 1% formaldehyde for 10 min. 3 × 10^6^ cells were lysed for 10 min. The chromatin was sheared through sonication performed ten times for 40 s, and then immunoprecipitated with the 2 g of antibodies targeting p53 (2524, CST), RNA polymerase II (ab300575, Abcam), SP1 (9389, CST), ETS‐1 (14069, CST) and STAT3 (9139, CST) overnight at 4°C. The crosslinking was reversed at 65°C overnight, and the samples were digested with 100 µL of proteinase K at 45°C for 1 h. DNA was then recovered for qPCR or semiquantitative PCR. The semiquantitative PCR products were analyzed through electrophoresis on a 1.5% agarose gel.

### 4.16 Statistical Analysis

Statistical analysis was performed using GraphPad Prism version 9.0. Differences among the groups were compared using a one‐way analysis of variance followed by Dunnett's multiple comparisons test or two‐tailed Student's *t*‐test. A *p*‐value of <0.05 was considered statistically significant.

## Author Contributions

Yanqi Dang performed most of the experiments and wrote the manuscript; Jing Ma performed the qPCR detection and immunofluorescence experiments, analyzed the data, and wrote the method section; Shuang Ling performed the immunofluorescence experiments, analyzed the data, and wrote the method section; Shurong Wang and Huining Guo performed the qPCR detection; Jun Liu performed some of the cell experiments and wrote the figure legends; Guang Ji developed design, provided laboratory support, revised the manuscript; J. Xu developed the concept and design, provided laboratory and financial support, wrote the manuscript, determine the final manuscript. All authors approved the final manuscript.

## Ethics Statement

All animal experiments in the study were approved by the Animal Ethics Committee of Shanghai University of Traditional Chinese Medicine (approval no. 201520001).

## Conflicts of Interest

The authors declare no conflicts of interest.

## Supporting information




**Supporting file 1**: mco270261‐sup‐0001‐SuppMat.pdf.

**Supporting file 2**: mco270261‐sup‐0002‐TableS1.xls.

**Supporting file 3**: mco270261‐sup‐0003‐TableS2.xls.

**Supporting file 4**: mco270261‐sup‐0004‐TableS3.xlsx.

**Supporting file 5**: mco270261‐sup‐0005‐TableS4.xlsx.

## Data Availability

The datasets used and/or analyzed during the current study are downloaded from the Genome Sequence Archive in the National Genomics Data Center, China National Center for Bioinformation/Beijing Institute of Genomics, Chinese Academy of Sciences (GSA‐Human: HRA011411) are publicly accessible at https://ngdc.cncb.ac.cn/gsa‐human.
