# Correction to “Tet Methylcytosine Dioxygenase 3 Promotes Cardiovascular Senescence by DNA 5‐Hydroxymethylcytosine‐Mediated Sp1 Transcription Factor Expression”

**DOI:** 10.1002/mco2.71013

**Published:** 2026-10-06

Y. Dang, J. Ma, S. Ling, ShurongWang, Huining Guo, Jun Liu, Guang Ji, Jin‐Wen Xu, “Tet Methylcytosine Dioxygenase 3 Promotes Cardiovascular Senescence by DNA 5‐Hydroxymethylcytosine‐Mediated Sp1 Transcription Factor Expression,” *MedComm* 6, 2025:e70261. https://doi.org/10.1002/mco2.70261.

We are submitting a request for a corrigendum concerning our published article titled “Tet Methylcytosine Dioxygenase 3 Promotes Cardiovascular Senescence by DNA 5‐Hydroxymethylcytosine‐Mediated Sp1 Transcription Factor Expression” (https://doi.org/10.1002/mco2.70261) in *MedComm*.

During a routine post‐publication review of our experimental data and figures, we discovered an error in the Western blot band for p21 in Figure 2E, wherein an incorrect band image was used. We have corrected this by replacing it with the correct original Western blot image of p21. We have meticulously re‐evaluated all raw data and quantitative statistical results associated with this figure and confirm that the statistical data remain accurate. Importantly, this image error does not impact any experimental results or the principal conclusions of the manuscript.

**FIGURE 2:**
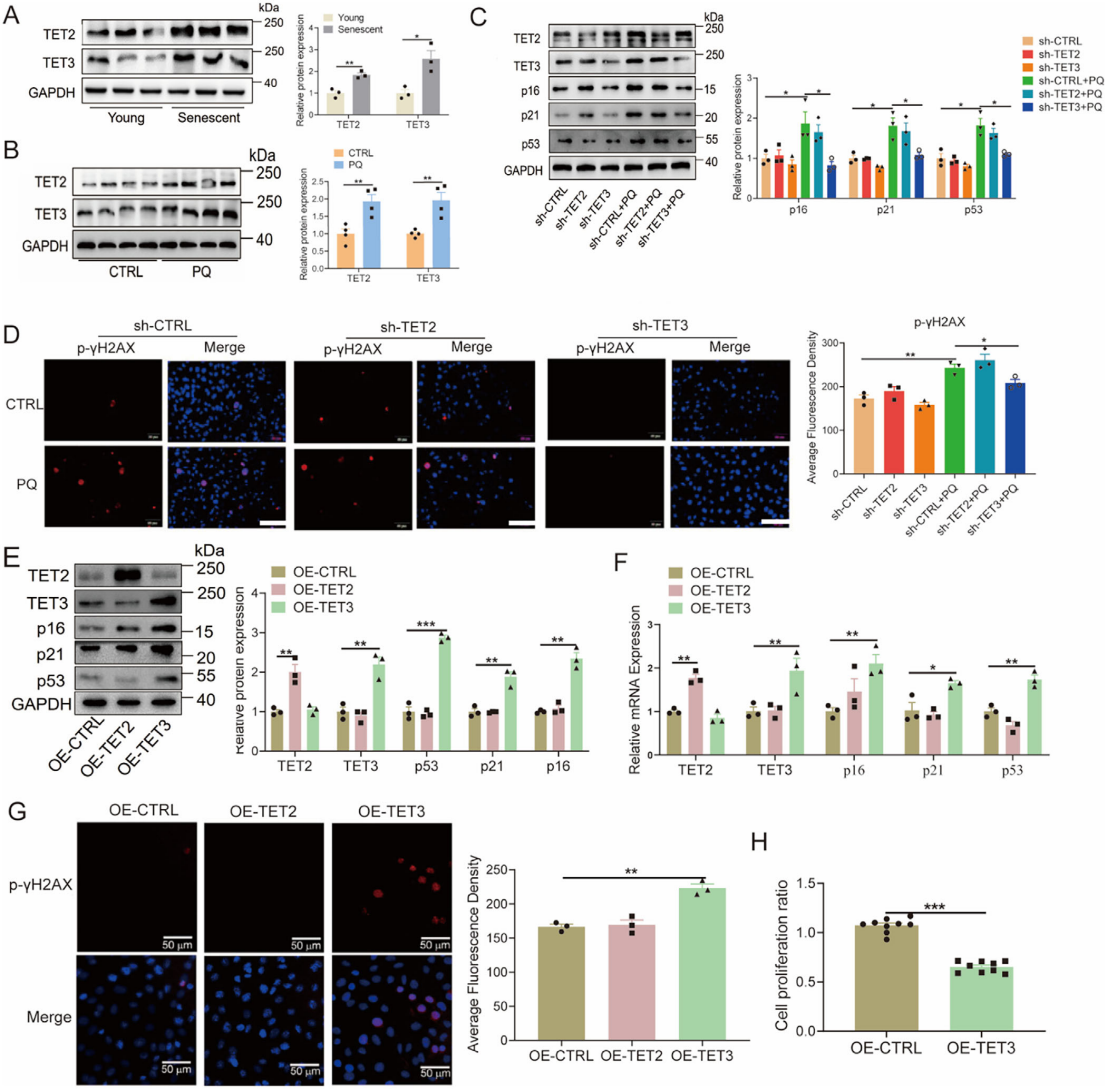
Role of TET3 in regulating cellular senescence. (A) Protein levels of TET2 and TET3 in replicative senescent HUVECs (*n* = 3 for each group, unpaired two‐sided *t‐*test). (B) Protein levels of TET2 and TET3 in HUVECs treated with PQ (*n* = 4 for each group, unpaired two‐sided *t*‐test). (C) Protein levels of p16, p21, and p53, and (D) Immunofluorescent imaging of p‐γH2AX after TET2 or TET3 silencing in HUVECs treated with PQ (*n* = 3 for each group, one‐way analysis of variance followed by Dunnett's multiple comparisons test, scale bars = 100 µm). (E and F) Protein and mRNA levels of p16, p21, and p53 after TET2 or TET3 overexpression (*n* = 3 for each group, unpaired two‐sided *t‐*test). (G) Immunofluorescent imaging of p‐γH2AX in HUVECs after TET2 or TET3 overexpression (*n* = 3 for each group, unpaired two‐sided *t*‐test, scale bars = 50 µm). (H) Cell proliferation after TET3 overexpression in HUVECs (*n* = 9 for each group, unpaired two‐sided *t*‐test). All the data are expressed as the mean ± SEM. ^*^ *p* < 0.05; ^**^ *p* < 0.01; ^***^ *p*< 0.001.

We extend our sincere apologies for this oversight and any inconvenience it may have caused to the journal and readers. All co‐authors have been informed and have consented to this correction. The revised Figure 2E is attached to this correspondence.

We apologize for this error.

