# Supplementary material for: Tet Methylcytosine Dioxygenase 3 Promotes Cardiovascular Senescence by DNA 5‐Hydroxymethylcytosine‐Mediated Sp1 Transcription Factor Expression
Source: MedComm (2020). 2025 Jun 19;6(7):e70261. doi: 10.1002/mco2.70261 (PMC12179407; doi:10.1002/mco2.70261)
Supplement: Supplementary file 1 — Supporting file 1: mco270261‐sup‐0001‐SuppMat.pdf. [file MCO2-6-e70261-s003.pdf]

Tet methylcytosine dioxygenase 3 promotes cardiovascular senescence by DNA 5-hydroxymethylcytosine-mediated Sp1 transcription factor expression

Yanqi Dang [1,2,3,#,*], Jing Ma [1,4,#], Shuang Ling [1,5], Shurong Wang [1], Huining Guo [1], Jun Liu [1], Guang Ji [2,3*], Jin-Wen Xu [1*]

1. Institute of Interdisciplinary Medical Science, Shanghai University of Traditional Chinese Medicine, 1200 Cailun Rd., Pudong New Area, Shanghai 201203, China
2. Institute of Digestive Diseases, Longhua Hospital, China-Canada Center of Research for Digestive Diseases (ccCRDD), Shanghai University of Traditional Chinese Medicine, Shanghai 200032, China
3. State Key Laboratory of Integration and Innovation of Classic Formula and Modern Chinese Medicine (Shanghai University of Traditional Chinese Medicine), Shanghai 200032, China
4. Seventh People's Hospital of Shanghai University of Traditional Chinese Medicine, No. 358 Datong Road, Pudong New Area, Shanghai 200137, China
5. School of Integrative Medicine, Shanghai University of Traditional Chinese Medicine, Shanghai 201203, China

Running Title: TET3 promotes cardiovascular senescence

[#] These authors have contributed equally to this work and share the first authorship.

[*]Address for correspondence:
Yanqi Dang, Email: dangyanqi9022@126.com and yq_dang@shutcm.edu.cn
Guang Ji, Email: jiliver@vip.sina.com and jg@shutcm.edu.cn;
Jin-Wen Xu, Email: jwxu1001@163.com;

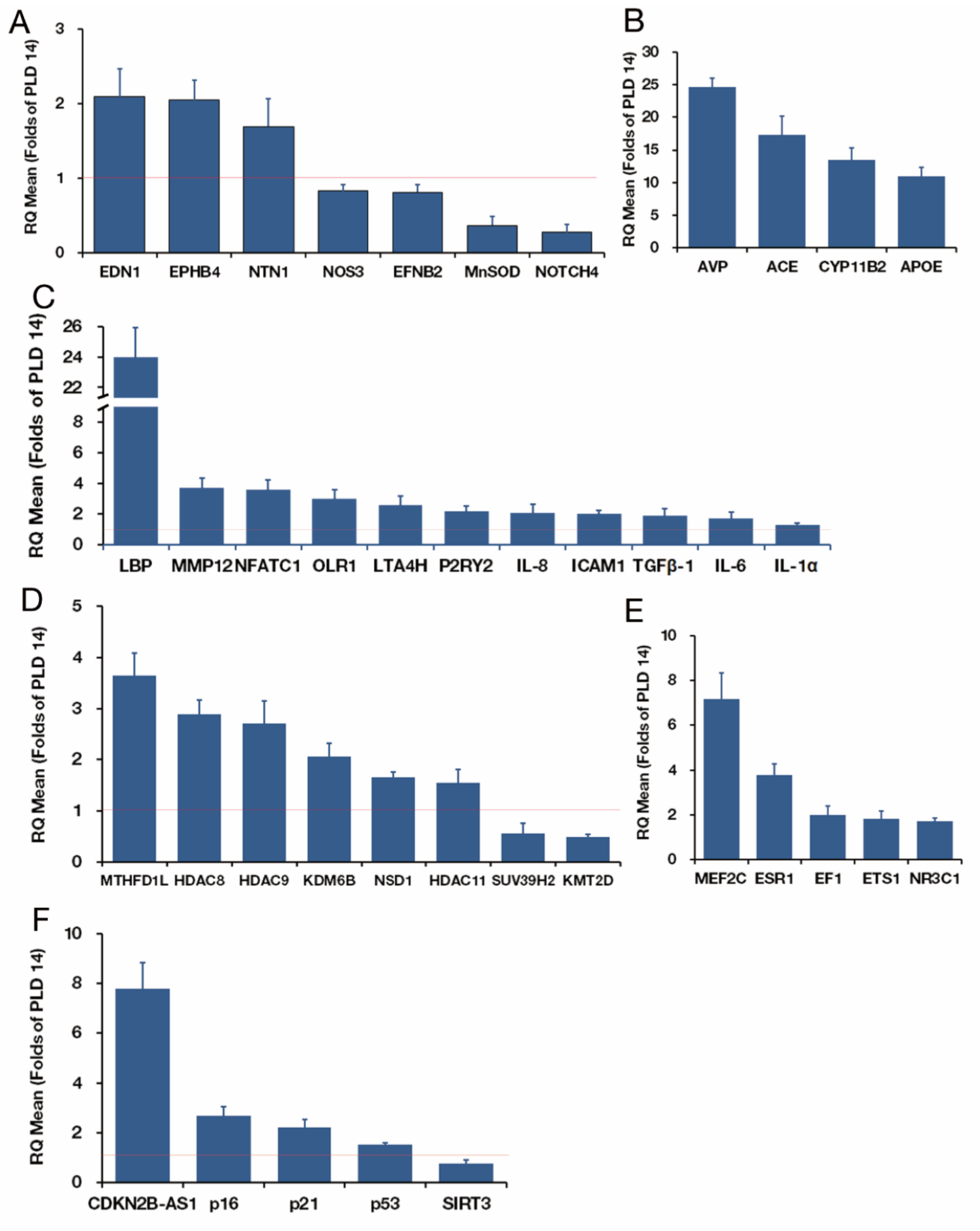

Figure S1 Expression of multiple genes involved in inflammatory cytokines, cardiovascular function, and epigenetic regulation in replicative senescent HUVECs.

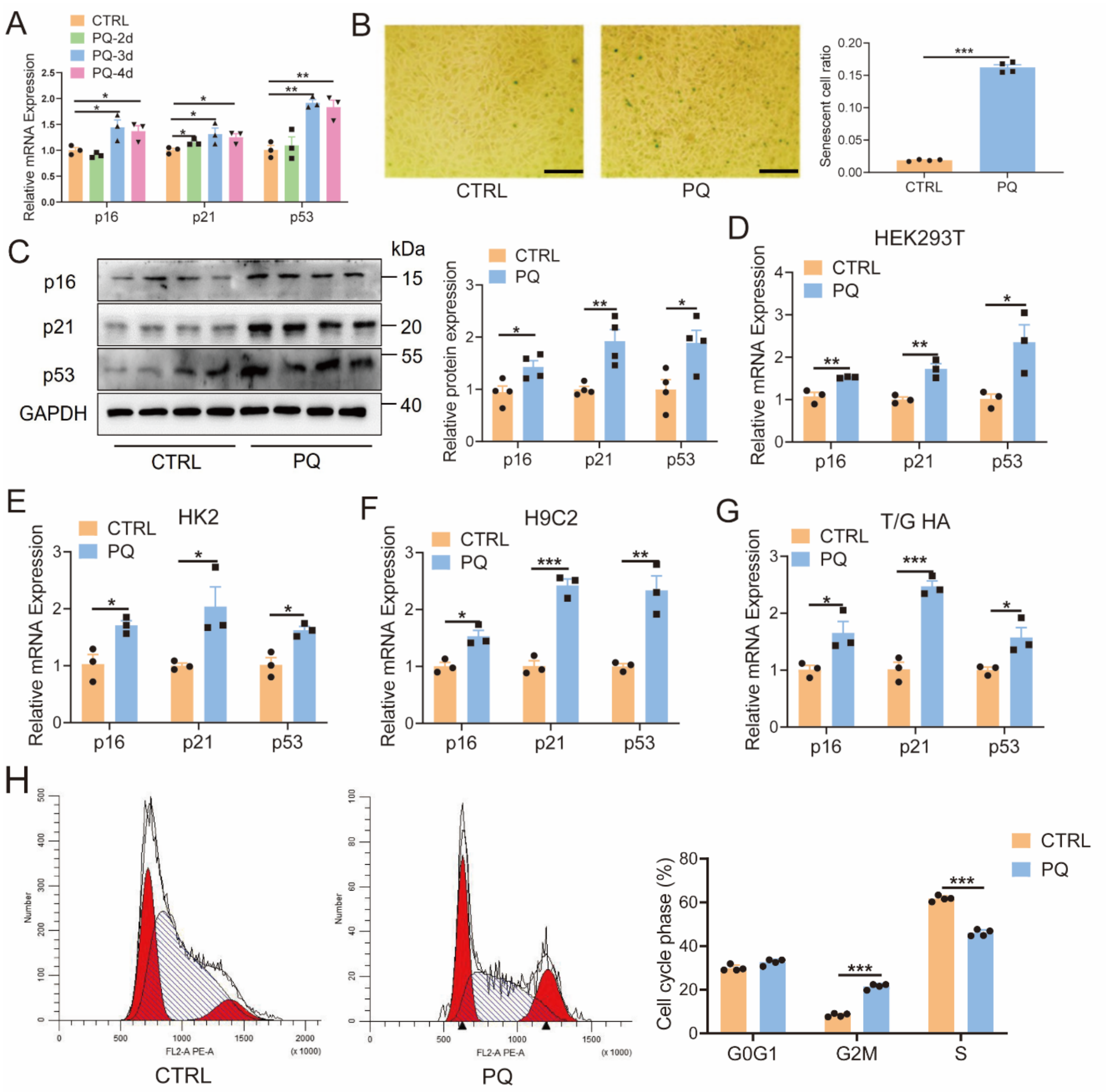

Figure S2 PQ induced cellular senescence in HUVECs. (A) mRNA levels of p16, p21, and p53 in HUVECs with PQ treatment for 2 days, 3 days and 4 days; (B) β-galactosidase staining and senescent cell ratio in HUVECs with PQ treatment for 3 days, scale bars = 100 μm; (C) Protein levels of p16, p21, and p53 in HUVECs with PQ treatment for 3 days; MRNA levels of p16, p21, and p53 in (D) HEK293T cells, (E) HK2 cells, (F) H9C2 cells and (G) T/G HA cells with PQ treatment for 3 days; (H) PQ significantly promoted the proportion of G2M stage cells and decreased the proportion of S stage cells. All data are expressed as the mean ± SEM. $^{*}P < 0.05$; $^{**}P < 0.01$; $^{***}P < 0.001$.

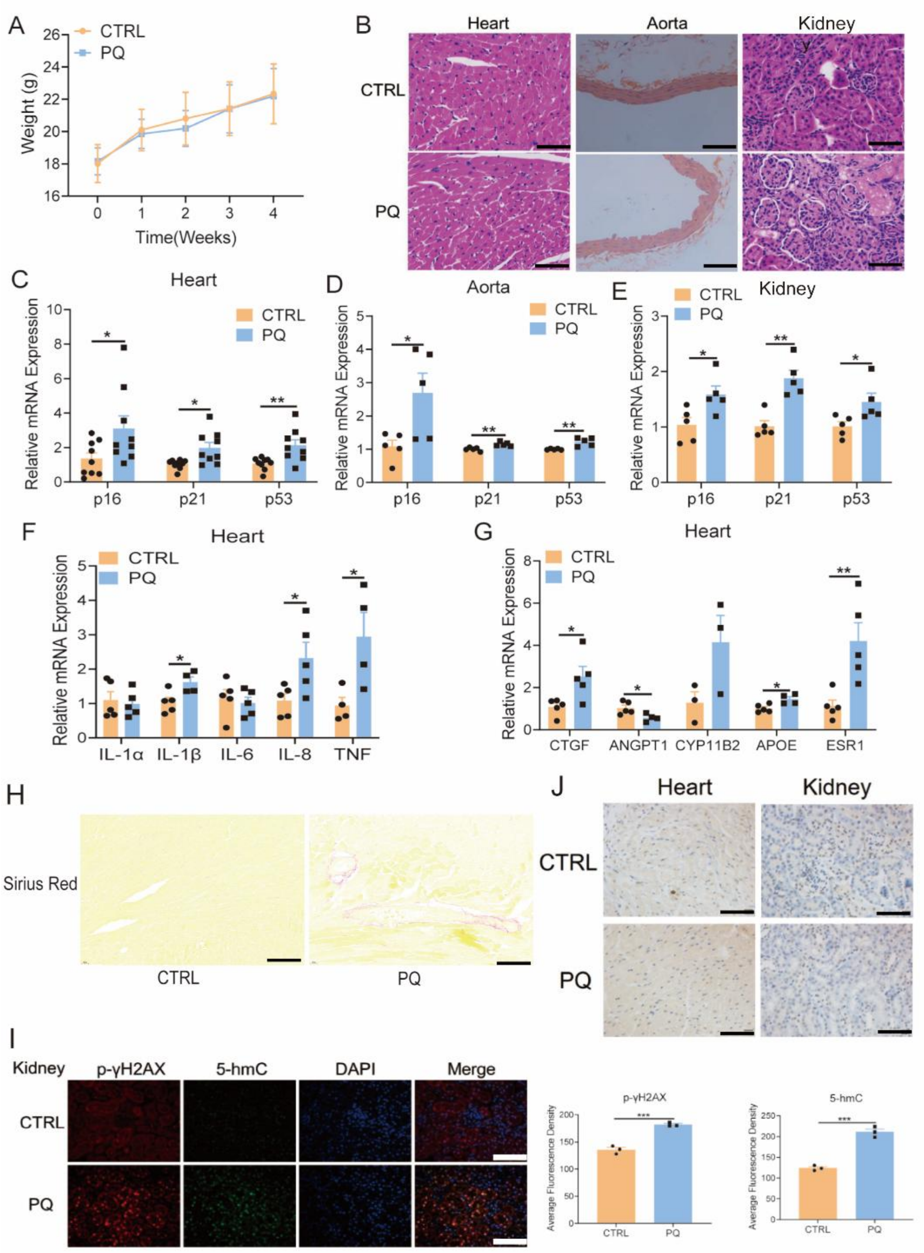

Figure S3 PQ induced aging in mice. (A) Weight of mice with PQ treatment for 4 weeks; (B) Hematoxylin-eosin (HE) staining in heart, aorta, and kidney of mice with PQ treatment for 4 weeks (scale bars = 100 μm); MRNA levels of p16, p21, and p53 in (C) heart, (D) aorta, (E) kidney of mice with PQ treatment for 4 weeks; (F, G) Levels of inflammatory cytokines, cardiovascular function, and epigenetic regulation in heart of mice with PQ treatment for 4 weeks; (H) PQ promoted myocardial fibrosis (scale bars = 100 μm); (I) Levels of p-γH2AX and 5-hmC in the kidney tissue of PQ-treated mice (scale bars = 100 μm); (J) TUNEL staining were performed in heart, and kidney of mice with PQ treatment for 4 weeks (scale bars = 100 μm). All data are expressed as the mean ± SEM. $^{*}P < 0.05$; $^{**}P < 0.01$; $^{***}P < 0.001$.

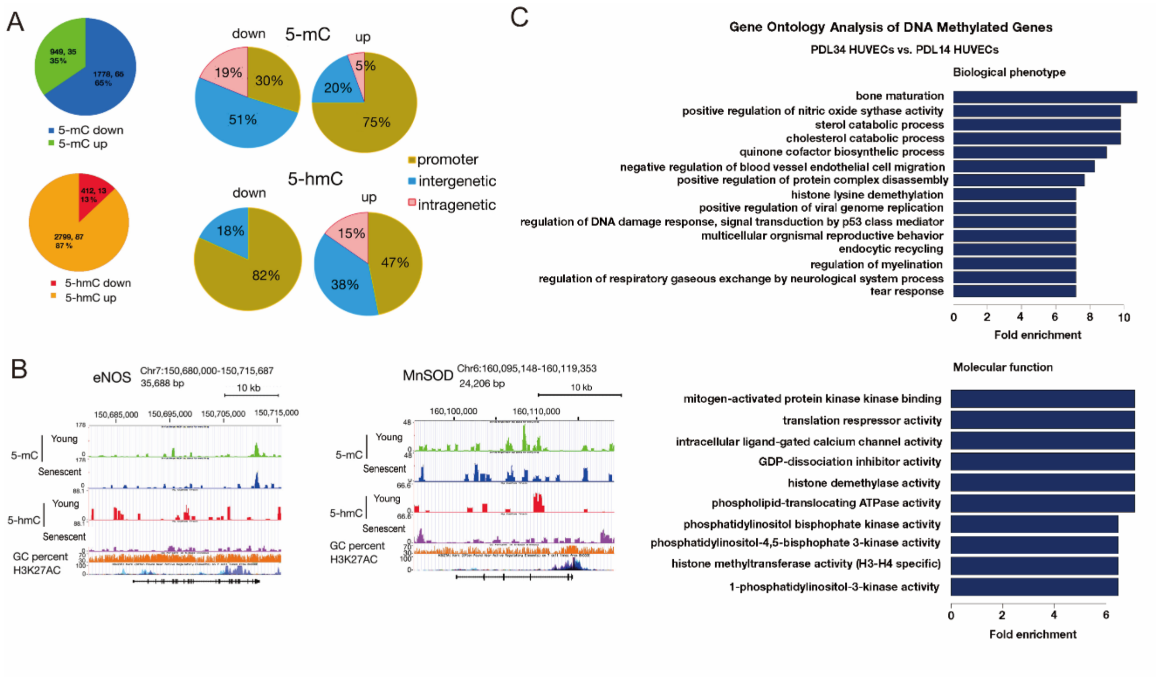

Figure S4 Analysis of 5-mC and 5-hmC between the replicative senescent HUVECs compared to the young HUVECs. (A) Changes of 5-mC and 5-hmC between the replicative senescent HUVECs compared to the young HUVECs; (B) The DNA 5-mC and 5-hmC levels of nitric oxide synthase 3 (eNOS), and superoxide dismutase 2 (MnSOD) between the replicative senescent HUVECs compared to the young HUVECs; (C) GO analysis of DhMGs were performed between the replicative senescent HUVECs compared to the young HUVECs.

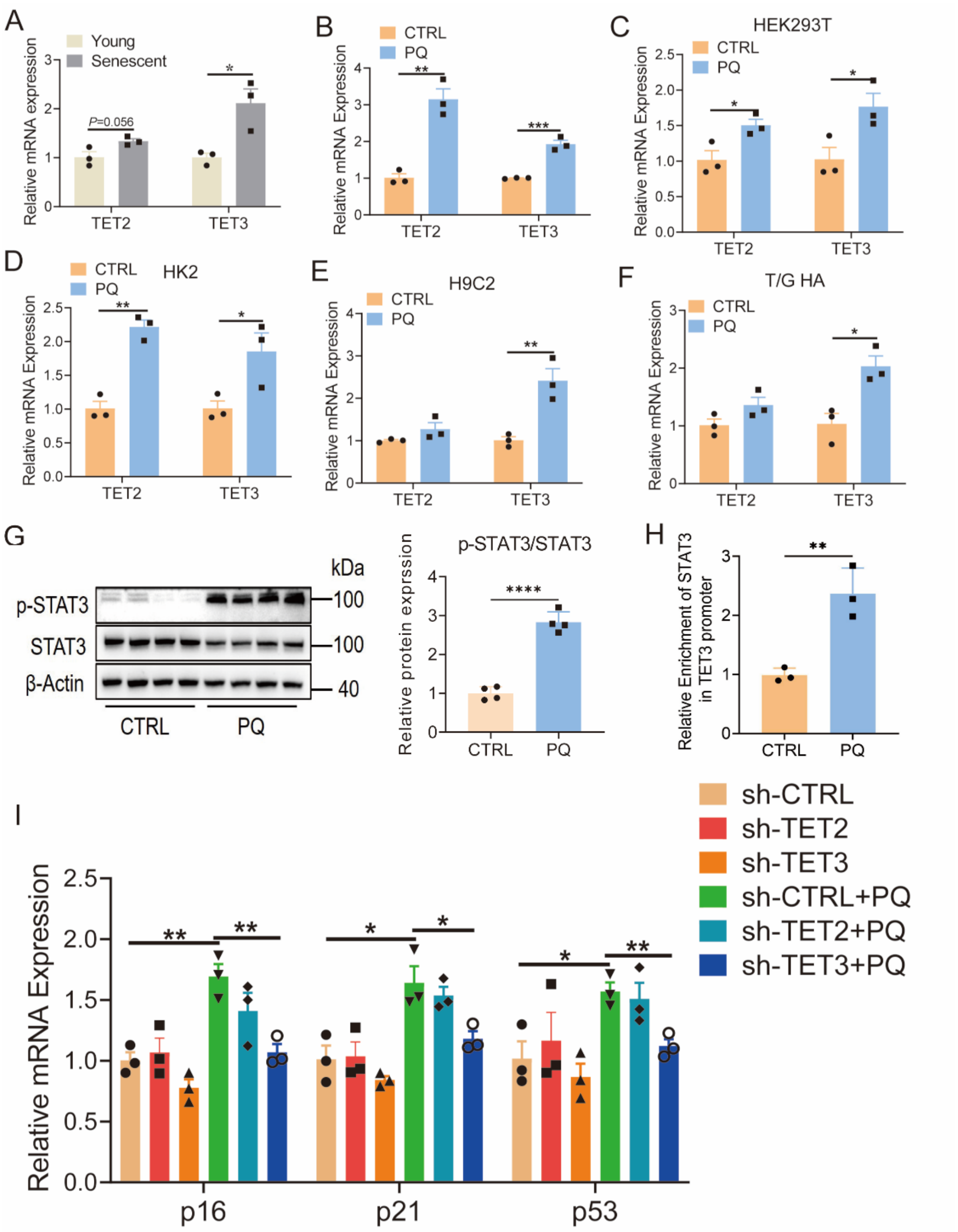

Figure S5 Role of TET3 in PQ-induced senescence and regulation of cellular senescence. MRNA levels of TET2 and TET3 in HUVECs with (A) replicative senescence and (B) PQ-induced senescence; MRNA levels of TET2 and TET3 in (C) HEK293T cells, (D) HK2 cells, (E) H9C2 cells and (F) T/G HA cells with PQ treatment for 3 days; (G) The levels of STAT3 and phosphorylated STAT3 after PQ treatment; (H) Enrichment of STAT3 in TET3 promoter was detected after PQ treatment; (I) MRNA levels of p16, p21, and p53 in PQ-induced HUVECs with TET2 or TET3 knockdown. All data are expressed as the mean ± SEM. $^{*}P < 0.05$; $^{**}P < 0.01$; $^{***}P < 0.001$; $^{****}P < 0.0001$.

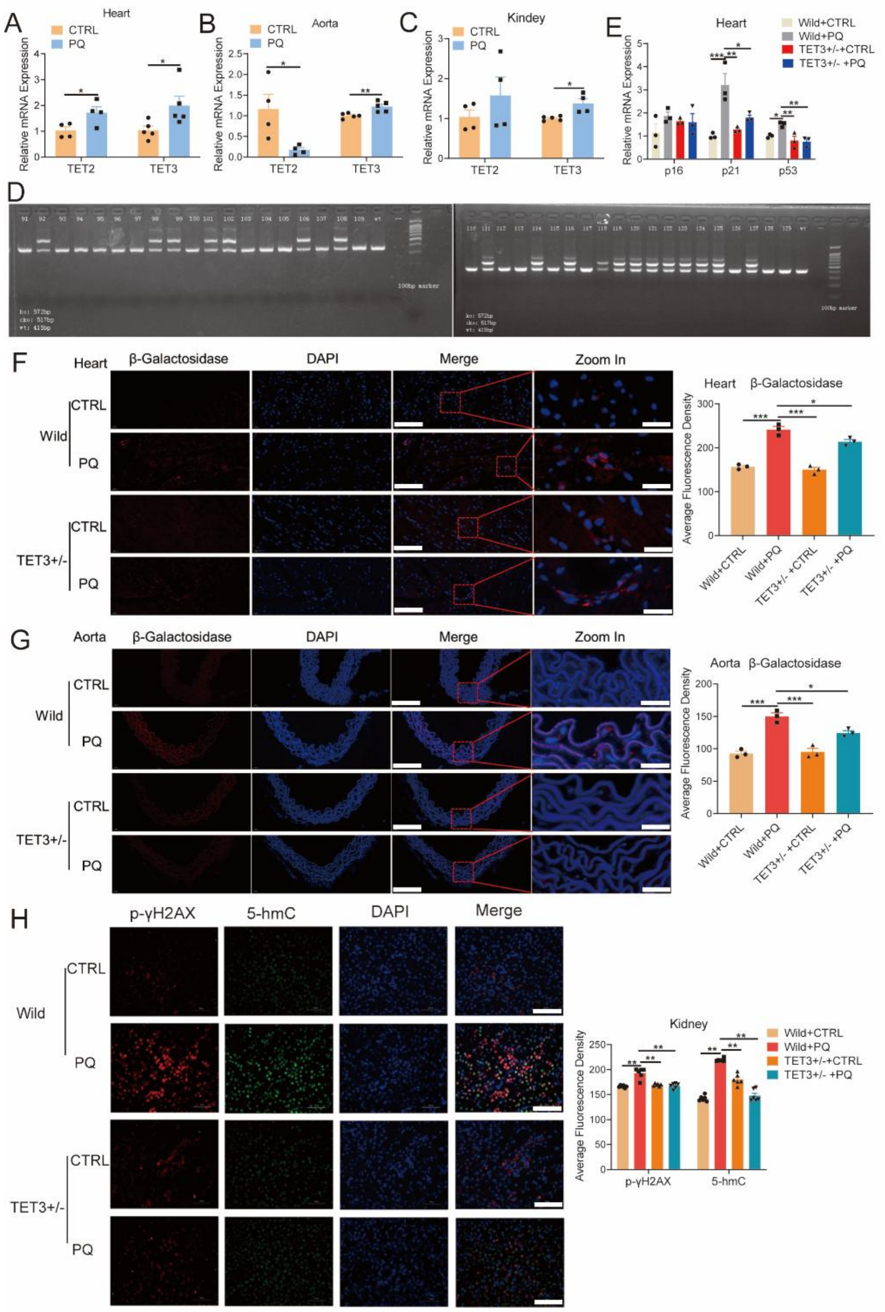

Figure S6 Role of TET3 on senescence in TET3-heterozygous mice. (A) MRNA levels of TET2 and TET3 in (A) heart, (B) aorta, (C) kidney of mice with PQ treatment for 4 weeks; (D) TET3-heterozygous mice were conducted; (E) MRNA levels of p16, p21 and p53 in the senescent heart tissues of TET3-heterozygous mice treated with PQ for 4 weeks; (F, G) Levels of β-galactosidase in the senescent heart and aorta tissues of TET3-heterozygous mice treated with PQ for 4 weeks (scale bars = 100 μm and 25 μm); (H) Levels of p-γH2AX and 5-hmC in the senescent kidney tissues of TET3-heterozygous mice treated with PQ for 4 weeks (scale bars = 100 μm). All data are expressed as the mean ± SEM. $^{*}P < 0.05$; $^{**}P < 0.01$; $^{***}P < 0.001$.

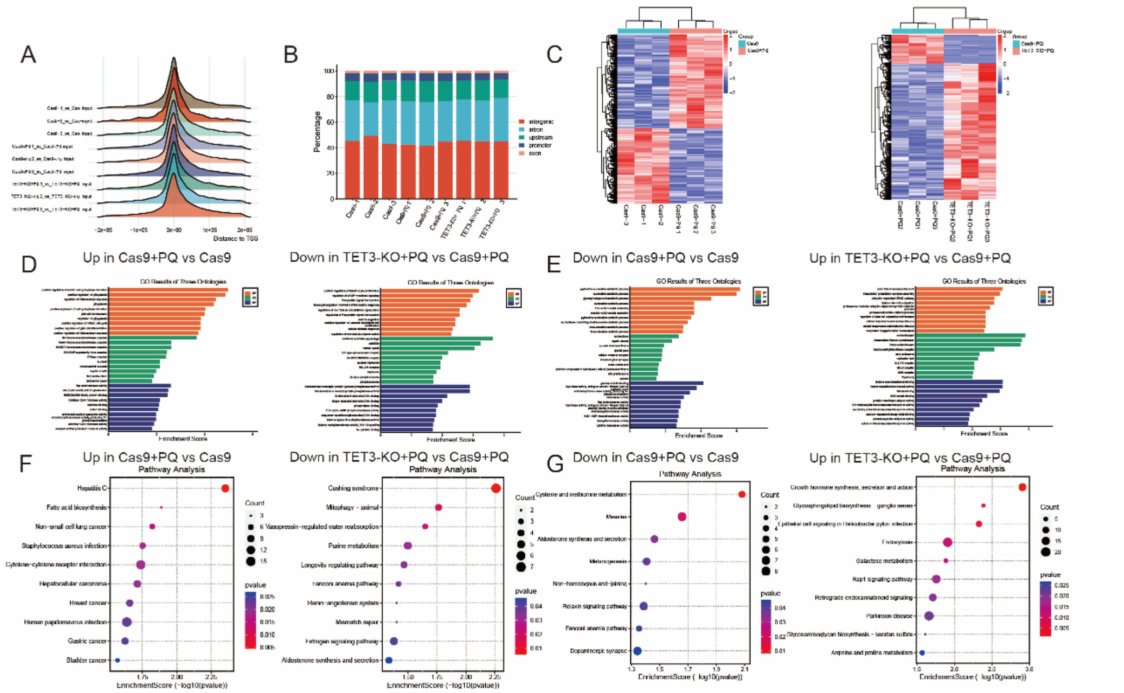

Figure S7 Analysis of 5-hmC in TET3 knockout/PQ-treated HUVECs based on hMeDIP-seq. (A) Location distribution of 5-hmC in TET3 knockout/PQ-treated HUVECs; (B) Distribution of 5-hmC in genome in TET3 knockout/PQ-treated HUVECs; (C) Heatmap of DhMGs among the three groups; (D, E) GO analysis of DhMGs among the three groups; (F, G) KEGG analysis of DhMGs among the three groups.

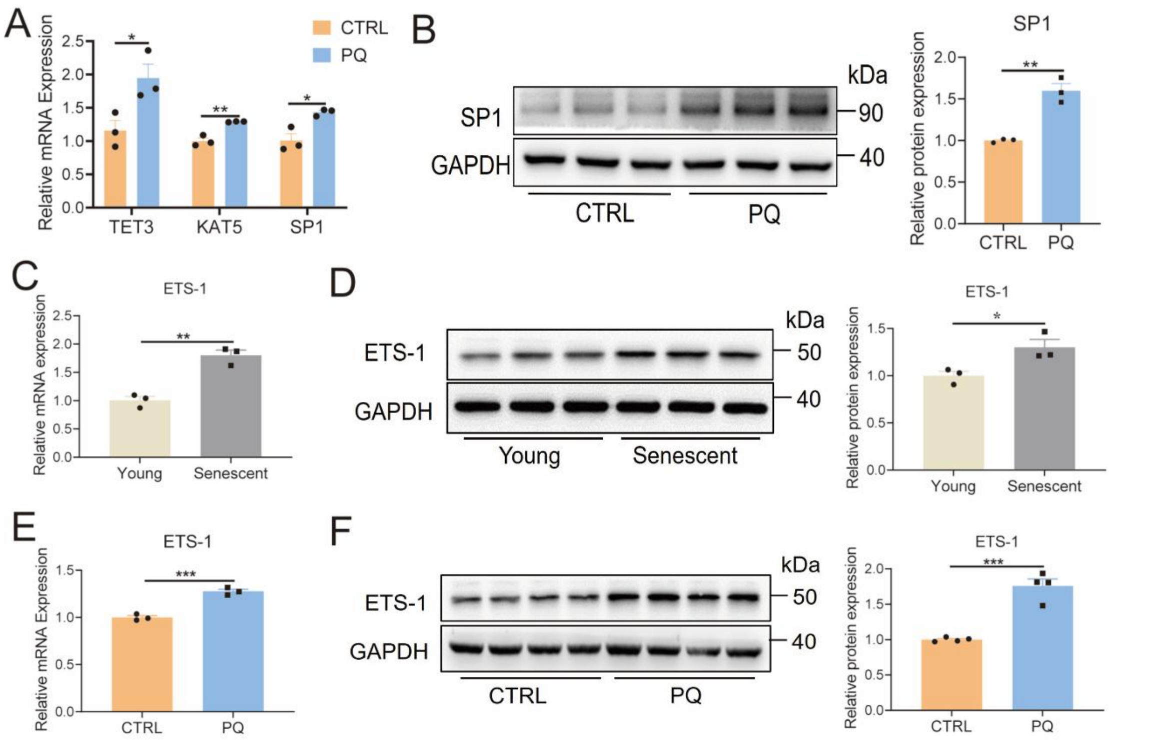

Figure S8 Levels of SP1 and ETS1 in senescent cells. (A) MRNA levels of TET3, KAT5, and SP1 in HUVECs with PQ treatment for 3 days; (B) Protein levels of SP1 in HUVECs with PQ treatment for 3 days; (C, D) MRNA and protein levels of ETS-1 in HUVECs with replicative senescence; (E, F) MRNA and protein levels of ETS-1 in HUVECs with PQ treatment for 3 days. All data are expressed as the mean ± SEM. $^{*}P < 0.05$; $^{**}P < 0.01$; $^{***}P < 0.001$.

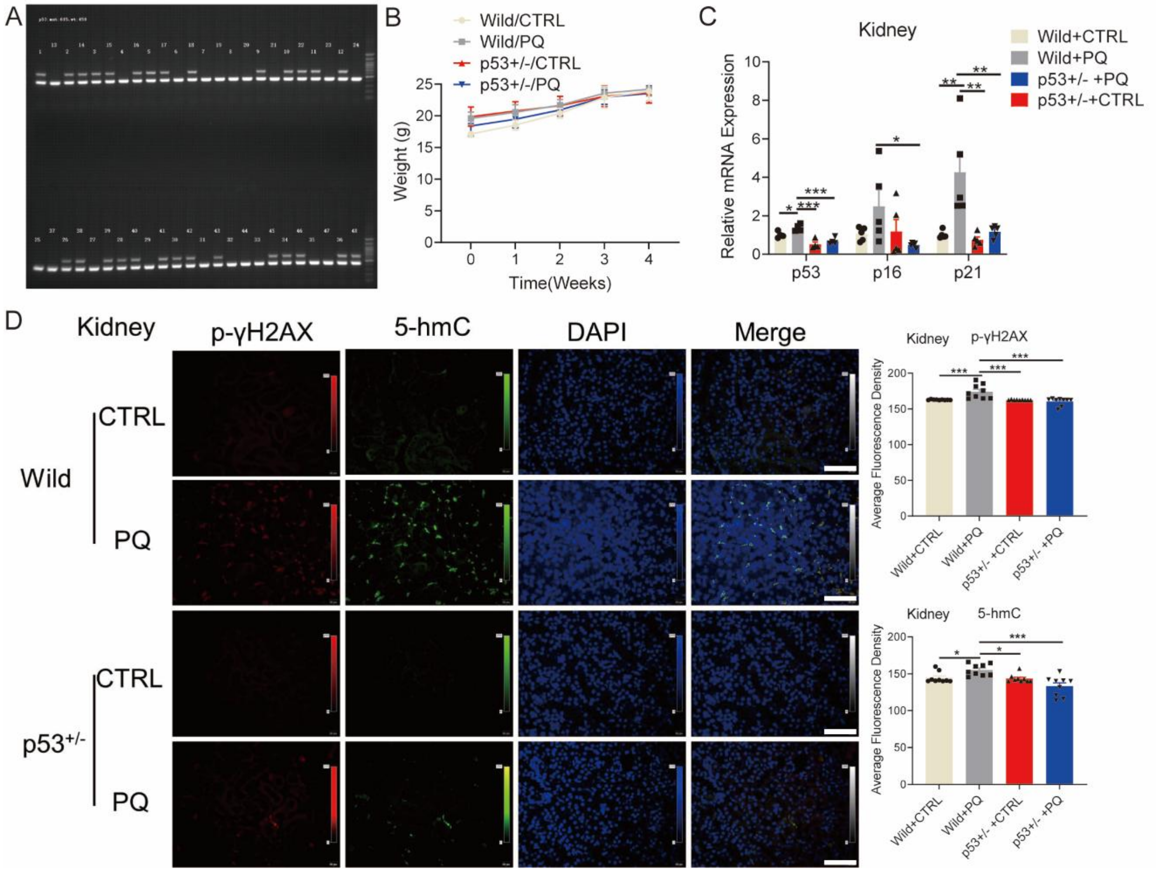

Figure S9 Role of p53 on senescence in p53-heterozygous mice. (A) p53-heterozygous mice were conducted; (B) Changes of weight in p53-heterozygous mice with PQ treatment for 4 weeks; (C) Levels of p53, p16, and p21 mRNAs in the kidney tissues of $p53^{+/-}$- heterozygous mice treated with PQ for 4 weeks; (D) Levels of p-γH2AX and 5-hmc in the kidney tissues of $p53^{+/-}$-heterozygous mice treated with PQ for 4 weeks (scale bars = 100 μm). All data are expressed as the mean ± SEM. $^{*}P < 0.05$; $^{**}P < 0.01$; $^{***}P < 0.001$.

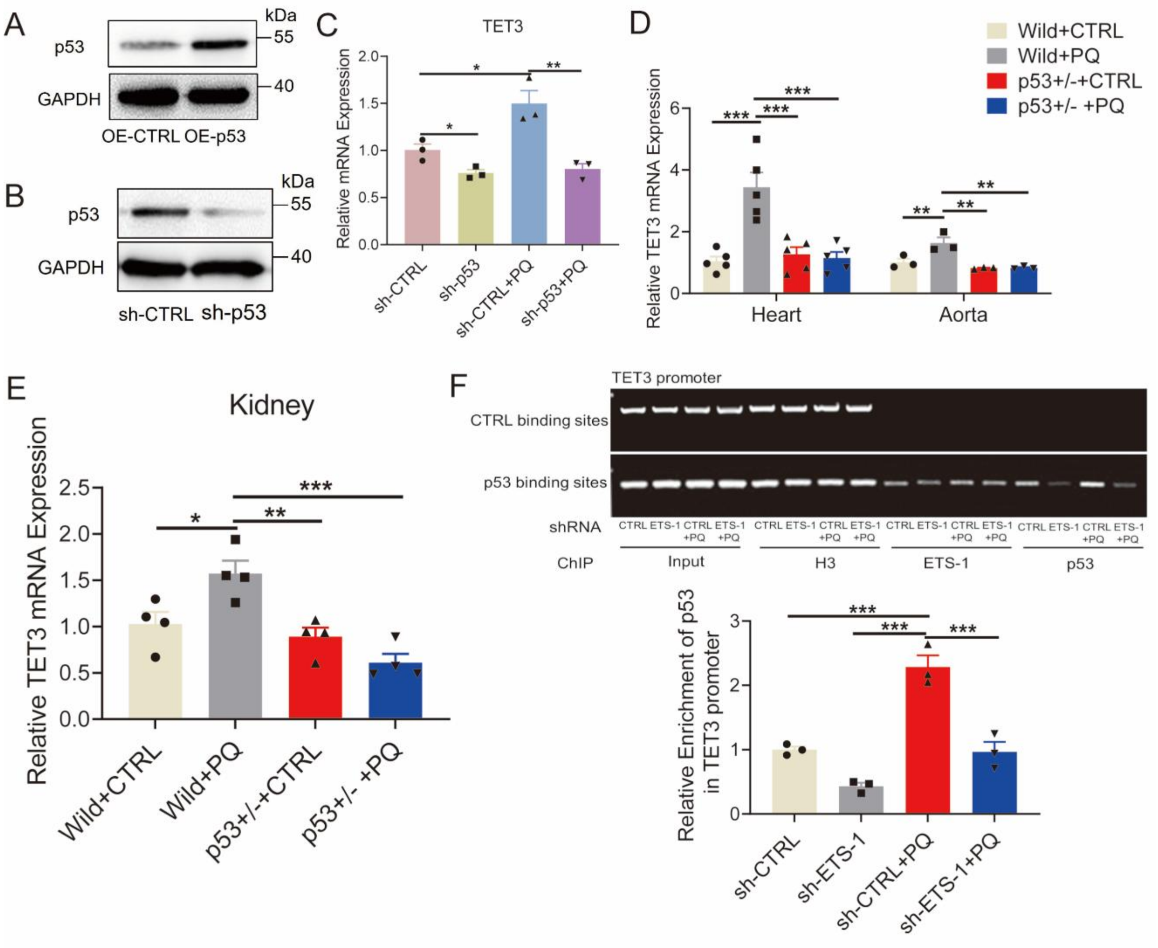

Figure S10 Regulation of TET3 expression by p53. (A, B) Overexpression and knockdown p53 were verified; (C) MRNA level of TET3 after p53 knockdown were shown; (D) Levels of TET3 mRNA in the heart and aorta tissues of $p53^{+/-}$-heterozygous mice treated with PQ for 4 weeks; (E) Levels of TET3 mRNA in the kidney tissues of $p53^{+/-}$-heterozygous mice treated with PQ for 4 weeks; (F) ETS-1 regulated the enrichment of p53 in the TET3 promoter. All data are expressed as the mean ± SEM. $^{*}P < 0.05$; $^{**}P < 0.01$; $^{***}P < 0.001$.

Table S5 The information of antibodies, primers and Reagents

REAGENT or RESOURCE	SOURCE	IDENTIFIER
**Antibodies**		
Anti-p16	Abcam	ab108349
Anti-p16	Santa Cruz	sc-1661
Anti-p21	Santa Cruz	sc-397, sc-6246
Anti-p53	CST	2524
Anti-TET2	Santa Cruz	sc-136926
Anti-TET3	Santa Cruz	sc-139186
Anti-TET3	Genetex	GTX-121453
Anti-GAPDH	KangChen Bio-tech, Shanghai	KC-5G5
Anti-SP1	CST	9389
Anti-ETS-1	CST	14069
Anti-STAT3	CST	9139
Anti-p-STAT3	CST	9145
Anti-β-actin	Huabio	1702-67
Anti-5-hmC	Abcam	ab106918
Anti-5-hmC	Active Motif	39769
anti-p-γH2AX	Abcam	ab26350
Anti-RNA polymerase II	Abcam	ab300575
Rabbit Anti-Mouse IgG H&L (Alexa Fluor® 488)	Abcam	ab150125
**Chemicals, Reagents, Plasmids**		
Paraquat	Yuanye Biotech, Shanghai	ZCM-GBW(E)060892
DAPI	Sigma	D9542
Irinotecan	Selleck	S2217
Doxorubicin	Selleck	S1208
Rapamycin	Selleck	S1039
Dulbecco's Modified Eagle Medium	Thermo Fisher Scientific	11960044
Ham's F-12K medium	Thermo Fisher Scientific	21127022
K-SFM medium	Thermo Fisher Scientific	10725018
RIPA lysis buffer	Beyotime Biotech, Nantong	P0013C
FuGENE® HD Transfection Reagent	Promega	E2311
TRIzol Reagent	Thermo Fisher Scientific	15596026
Power SYBR Green PCR Master mix	Thermo Fisher Scientific	4367659
pCDH-CMV-MCS-EF1-Green Puro plasmid	SBI	CD513B-1
pRL-SV40 plasmid of renilla luciferase gene	Promega	E2231
Luc pGL3-basic plasmid	Promega	E1751
**Critical Commercial Assays**		
Cell Counting Kit-8	Beyotime Biotech, Nantong	C0038
Dual-Luciferase Reporter Assay	Promega	E1910
Cellular Senescence Assay	Cell Biolabs	CBA-230

Chromatin immunoprecipitation assay	Abcam	ab500
Tunel Detection Kit	Roche	11684817910
High-Capacity cDNA Reverse Transcription kit	Thermo Fisher Scientific	4368814

Experimental Models: Cell lines

HUVEC	ATCC	CRL-1730
VSMC	ATCC	CRL-1999
H9c2	Cell Bank of Chinese Academy of Sciences, Shanghai	GNR 5
293T	Cell Bank of Chinese Academy of Sciences, Shanghai	SCSP-502
HK-2	Cell Bank of Chinese Academy of Sciences, Shanghai	SCSP-511
EA.hy926	Cell Bank of Chinese Academy of Sciences, Shanghai	GNHu39

Experimental Models: Organisms/Strains

C57BL/6J	Shanghai SIPPR-BK Laboratory	N/A
TET3-heterozygous	Shanghai Model Organisms Center	N/A
p53-heterozygous	Shanghai Model Organisms Center	N/A

shRNA plasmid

TET2	SCBT	sc-88934-SH
TET3	SCBT	sc-94636-SH
p53	SCBT	sc-29435-SH
ETS-1	SCBT	sc-29309-SH
Control shRNA plasmid-A	SCBT	sc-108060

CRISPR/Cas9 Gene

TET3 CRISPR/Cas9 KO Plasmid(h)	SCBT	sc-414997
TET3 HDR Plasmid(h)	SCBT	sc-414997-HDR
Control CRISPR/Cas9 Plasmid	SCBT	sc-418922

Software and Algorithms

GraphPad Prism v9.0	http://www.graphpad.com/	N/A
Image J	https://imagej.nih.gov/ij/	N/A

Primers sequences used in real-time qPCR

SPECIES	TARGET GENE	FORWARD PRIMER (5'->3')	REVERSE PRIMER (5'->3')	SOURCE
Human	ACE	CCAGGTGGTGTGGAACGAGTATG	CAGGGTGTGGTTGGCTATTTGC	Generay
Human	ANGPT1	GAAAACACAAGGAAGAGTTGGACAC	GAAGGACACTGTTGTTGGTGGTAG	Generay
Human	APOE	CACAGGCAGGAAGATGAA	AGGTAATCCCAAAAGCGA	Generay
Human	AVP	CATACGGGGTCCACCTGTGTG	TCGGGCAGTTCTGGAAGTAGCAC	Generay
Human	CASP1	TCGGAATAACGGAGTCAATCA	ACAGACAAGGGTGCTGAACAA	Generay
Human	CASP5	TGTCAAGGTTGCTCGTTCTAT	AGGACTTCATTGCTTTCTGTTC	Generay
Human	CDKN2B	GATCCCAACGGAGTCAAC	GTGAGAGTGGCAGGGTCT	Generay
Human	CDKN2B-AS1	TGGGGATACGAAGCTCTACACACT	GATCACAGACCATACTTGCACTGG	Generay
Human	CEBPA	GGATAACCTTGTGCCTTGGAAATGC	TTCTCTCATGGGGGTCTGCTGTAG	Generay
Human	CTGF	CCTGCATCTTCGGTGGTACGGT	ACACCCACTCCTCGCAGCATTT	Generay
Human	CYP11B2	AACTTGGGAGGACCACGCAT	GGCCCATTCAACAAGAACACG	Generay
Human	EDN1	GGAACACCTAAGACAAACCA	GGGTCACATAACGCTCTC	Generay
Human	EFNB2	TCCAAAAAGAACAATAATGAGAGCA	CCAACGCAGAAATAAACGCC	Generay
Human	eNOS	TGGAGAATGAGCAGAAGG	ATGGAAAACAGGAGTGAGG	Generay

Human	EPHB4	CGGACAAACACGGACAGTATCTC	CCTGCACCAATCACCTCTTCAA	Generay
Human	ETS-1	GAGTCAACCCAGCCTATCCA	GGGCTCTGAGAACTCCGATG	Generay
Human	FCGR1A	GCCTTGAGGTGTCATGCGTG	GATATTCCTGCTGATGTGTAGCGAT	Generay
Human	F2RL2	TGGTATGTTAAGGCGAGTCTCC	AGCCATCAGTGTTGTTGTAGTAGTA	Generay
Human	GAPDH	CGCTGAGTACGTCGTGGAGTC	GCTGATGATCTTGAGGCTGTTGTC	Generay
Human	HDAC8	AGTTTTGACCCTACTTATTTGTGCC	CTCCTATTCTTGAGATCGCCG	Generay
Human	HDAC9	TGGAACCAGTGAAAAGGAAAGC	AACAGAAGGCAAGAAGGGAAACATA	Generay
Human	HDAC11	GCACCTAACATCCATTCACACCC	ATTCTTTGCCTCCAGCACACACA	Generay
Human	HMGCK	GCTTGTGTGTCCTTGGTATT	CGGCTTATTTTCTTCTTCTTC	Generay
Human	ICAM1	CCTGATGAGAGGGGAAGTGGTG	GAAGTGTGGGCCTTTGTGTTTTG	Generay
Human	IL-1α	GCCCAAGATGAAGACCAACCAGT	CCGTGAGTTTCCCAGAAGAAGAGG	Generay
Human	IL-1β	AAACAGATGAAGTGCTCCTTCCAGG	TGGAGAACACCACTTGTTGCTCCA	Generay
Human	IL-6	CCTCCAGAACAGATTTGAGAGTAGT	GGGTCAGGGGTGGTTATTGC	Generay
Human	IL-8	GTGTAAACATGACTTCCAAGCTG	TGGTCCACTCTCAATCACTCTC	Generay
Human	KDM6B	ACGAGTCAGAGCACGATAGTGAG	GAGCCAGTATGAAAGTTCCAGAG	Generay
Human	KDR	CAGAGTGAGGAAGGAGGAC	CAAGAAGTAGCCAGAAGAACA	Generay
Human	KMT2D	GGAGGAGGAAGAAGATGATGACACC	GCACTGCGAACAGGCAAGGA	Generay
Human	LBP	AAGGGCATCAGCATTTCGGTC	TTCAACAGCCACCCCAAGTCTC	Generay
Human	LTA4H	ATGCCCGAGATAGTGGATACCT	CCTGAGACTGGACCGTGAGAG	Generay
Human	MEF2C	GAGAGAGAGAAGAAAAACGGGGA	GCTTGTTGGTGCTGTTGAAGAT	Generay
Human	MMP12	TGGACTACACATTCAGGAGGCACAA	GTATGTCATCAGCAGAGAGGCGAAA	Generay
Human	MnSOD	GTGACTTTGGTTCCTTTGAC	GTGTCCCCGTTCCTTATT	Generay
Human	MTHFD1L	CGACGTTTGGAGTGAAAGGAGG	GCAAGTTATTGGCAGCGGTGAT	Generay
Human	mTOR	TCAGCGAGTTCTTGCTATTCC	AAAACCTCGTCACATTACCCC	Generay
Human	NFATC1	ACTACTCCTCTTTCCAGCACATCA	GCACCTCAATCCGAAGCTCATAC	Generay
Human	NLRC5	TACTCAGCAGCAGCACCTCCA	GCTCACCAAAGACCCAGAATG	Generay
Human	NOTCH4	ACTCTCGGTGCTGGCTTCTCTT	CTGGAGGGGTCTCACAGTCGTAG	Generay
Human	NR3C-1	GTTTCTGCGTCTTCACCCTCACT	CATTTCCCATCACTTTTGTTTCTGT	Generay
Human	NSD1	CAACCCATTGCCACGGAAGAAAA	CCAGCATCCCCACAACTAAAACA	Generay
Human	NTN1	GCTGCCTTTCCTATCCACACACC	ACACCTCAATTCCCATTTCCCTC	Generay
Human	OLR1	TTATCACCTTCCCCTCACACTCC	CCTCTGTCCTTCTTTGTTTCTTCAC	Generay
Human	p16	TTTTCACTGTGTTGGAGTTTTCTGG	TGAGCTTTGGTTCTGCCATTTG	Generay
Human	P2RY2	GTCCCACGCACCCTCTCATCTAT	TCCTCACTTCTCACGCCCTTCTC	Generay
Human	p21	AGCGACCTTCCTCATCCACC	AAGACAACTACTCCCAGCCCCATA	Generay
Human	p53	AGCGACCTTCCTCATCCACC	AAGACAACTACTCCCAGCCCCATA	Generay
Human	PAFAH1B3	GAGAAGAACCGACAGGTGGTGAACGAG	CGGCAAACAGGTGTGTAGCC	Generay
Human	RB1	CACCAAGGTCCTGAGATCCTC	AGCAACCCTCCTAAACCACTG	Generay
Human	SIRT1	TGAATTGTGTCATAGGTTAGGTGG	TTTTGTGTTCGTGGAGGTTTT	Generay
Human	SIRT3	TGGAAACTACAAGCCCAA	ACACTCTCTCAAGCCCATC	Generay
Human	SUV39H2	ATGGAAAACAGGAGTGAGG	CATACTGTTCTGACCCTCTTTT	Generay
Human	TET1	CTCTTGGTATGAGTGGGAGTGTTGT	GTTTGATGATGATTGAGGAGTGTGT	Generay
Human	TET2	CGTAGAGAAGCAGAAGGAAGCAAGA	ACAGGAGCAAAGGCAAGTAAACAAT	Generay
Human	TET3	CCTCCAGAACAGATTTGAGAGTAGT	GGGTCAGGGGTGGTTATTGC	Generay
Human	TGFβ-1	GACAGCAGGGATAACACACT	ATGAGAAGCAGGAAAGGC	Generay
Human	TLR4	CTAAACCAGCCAGACCTTGAA	AGACCTGTCCCTGAACCCTAT	Generay
Human	TLR9	TGAGTGCTCGTGGTAGAGGTCC	GCAGTCAATGGCTCCCAGTTC	Generay
Human	TP53BP1	AAGTTAGAAGAATCCACAGGGT	AGAACGAGGAGACGGTAATAGT	Generay
Human	TP53BP2	CATTCCCAGTGCTTTGAGGTA	GAAAATACAGACACTGCCCAACA	Generay
Human	UNC5D	GAGACACAATGGAGAGGTAAGACAG	GGGTAAGGAAGAGGAAGAAAGAATA	Generay
Human	VEGFA	ACGGACAGACAGACAGACA	CGAGAACAGCCCAGAAGT	Generay
Human	DOK5	AACAGGTTCCAAGAAGGAGAT	TAATGACATAGTGAAGCAAGGG	Generay
Human	KLF4	GGTGCTTGGTGAGTCTTGGTTC	CTTAGGTCATAAATGTTGATCGGA	Generay
Human	ATF7	AGATGGGAGGGGATAAGATGA	CCAAAGGACAGAACTGAGCAT	Generay
Human	FGF10	TCCTTCGTGTTTTCTGTCCTC	TTTAACTGGCAGCATAATGGG	Generay
Human	MET	CACATTTTCCTTGGTGCCACTAAC	CCTGATAAATTGGCTTTGCTGCT	Generay
Human	CDKN2C	GGCAGGTTCCCTTCATTATCC	AACTGGTTTCGCTGTCATTCA	Generay
Human	MAPK8	ATGTTGCCTCTCCGTATCCTGTT	CCCTTTACCTCTTCATCTTTCTTGC	Generay
Human	MAPK9	GCAGTCAAGAAACTAAGCCGT	GTGGTGTAAACACATTTAACAAA	Generay
Human	MAPK11	CTCACAGTCCTCGTTCACAGC	TACTTGGTGACCACCCTGATG	Generay
Human	MAPK12	TGGTCAGGATAGAGGCAAAAT	CTACACGCAGACGGTGGACAT	Generay
Human	MAPK14	AAGGAAGGAGGCAGACTGATGG	CTGTGGATGGTGAGGATTTGAAC	Generay
Human	EIF4EBP1	CCGCTTATCTTCTGGGCTATT	ACTCACCTGTGACCAAAACAC	Generay

Human	Casp9	GAGGATGACCACCACAAAGCA	AAGAACGACCTGACTGCCAAGA	Generay
Human	IL-1R1	TTTTACTCCGAAGAAGCTCAC	GAAACTCAACTTACTGCCTCAA	Generay
Human	TLR5	CAGTCTTCGGTGGATATGTTGT	CTTTGTTTTCTTCGCTTCGTG	Generay
Human	IL10	GCTCTTATTTTCACAGGGGAG	GCTGGACAACATACTGCTAACC	Generay
Human	IL18	TACAGGCGAGGTCATCACAA	GGGGATAAATCTGTAATGTTCACTC	Generay
Human	SP1	GTGGAGGCAACATCATTGCTG	GCCACTGGTACATTGGTCACAT	Generay
Human	KAT5	AACAAACGTCTGGATGAATGGG	AGGAAGTCCGTTCTTAGTGGG	Generay
Human	PRKAA1	TTGAAACCTGAAAATGTCCTGCT	GGTGAGCCACAACTTGTTCTT	Generay
Mouse	ANGPT1	ATTCTTCGCTGCCATTCT	GCTCTGTCGCACTCTCAC	Generay
Mouse	APOE	CAAGATGGAGGAACAGACC	TGATGATGGGGTTGGTAG	Generay
Mouse	CYP11B2	GCTGGAGAGGAAAAGGAG	CTGGGCATCAAAACAAAG	Generay
Mouse	CTGF	ACCGACTGGAAGACACATT	GGTATTGTCATTGGTAACTCG	Generay
Mouse	ESR1	GGTGCCCTACTACCTGGA	CTTTCTCGTTACTGCTGGAC	Generay
Mouse	GAPDH	CGCTGAGTACGTCGTGGAGTC	GCTGATGATCTTGAGGCTGTTGTC	Generay
Mouse	IL-1α	GACCGACCTCATTTTCTTCT	GCACCCGACTTTGTTCTT	Generay
Mouse	IL-1β	TCGTGAATGAGCAGACAG	AGAGGCAAGGAGGAAAAC	Generay
Mouse	IL6	AACCACGGCCTTCCCTACT	CATTTCCACGATTTCCCAGA	Generay
Mouse	IL8	GTCCGTCCCTGTGACACTCA	CCAACAGTAGCCTTCACCCAT	Generay
Mouse	KDR	TGTGGTCTTTCGGTGTGT	TGGGGTAGTGTAGTCAGGAG	Generay
Mouse	MnSOD	AGGTCGCTTACAGATTGCT	CTCCCAGTTGATTACATTCC	Generay
Mouse	p16	GCAGCTCTTCTGCTCAACTACG	TTGATGTCCCCGCTCTTGG	Generay
Mouse	p21	TACTTCCTCTGCCCTGCTGC	GCTGGTCTGCCTCCGTTTT	Generay
Mouse	p53	GGAGACATTTTCAGGCTTATGG	ACACTCGGAGGGCTTCACTT	Generay
Mouse	SIRT1	CTGGGGTTTCTGTCTCCT	AATGGTCTTGGGTCTTTTCT	Generay
Mouse	SIRT3	CCTGAAGCCATCTTTGAAC	GAAGTAGTGAGTGACATTGGG	Generay
Mouse	TET2	CCCTCAACCATCGCTTACCT	TGGCATTATCAGCATCACAGG	Generay
Mouse	TET3	GCCGGAAGCATCTCAGAATG	TGTCGGGAACAATGGTAGGG	Generay
Mouse	TNFα	CCCCTTTATTGTCTACTCCTC	CCCAGCATCTTGTGTTTC	Generay
Rat	p16	GCGGTATTTGCGGTATCT	CAGAAGTGAAGCCAAGGA	Generay
Rat	p21	GGGCTTTCTTTGTGTATTTG	CCTGTTTCGTGTCTACTGTTC	Generay
Rat	p53	CTACCCGAAGACCAAGAAG	CATAAAATGGCAAGAAAGGA	Generay
Rat	TET3	ATAGGTGGTGTGGCTGTG	TGAGGTTCTTGTGCTGGT	Generay
Rat	TET2	ATGGTATGGGCAGTAGGG	CGCAAGAGAAGTCGGTAG	Generay

Primers sequences used in ChIP assay

**SPECIES**	**GENE**	**Transcription factor**	FORWARD PRIMER (5'->3')	REVERSE PRIMER (5'->3')
Human	p53	SP1 and ETS-1 in binding site of p53	TGAACGCTTCTATCTTGGC	GAAGCCTGGAGAATGAGAT
Human	TET3	p53 in binding site of TET3	CCACCTCCTGAGTTTGTCC	GCTTTCCAGCTACCAGACA
Human	TET3	p53 in non-binding site of TET3	TTTACATAGTTGGCAGTC	CACTCATCTAAACCTGCA
Human	TET3	STAT3 in binding site of TET3	GTCCTGTGGCTGTTTCTGC	AACTGCCTTCCACCTACCTC
